# Pancancer analysis of DNA damage repair gene mutations and their impact on immune regulatory gene expression

**DOI:** 10.1038/s41598-025-99965-y

**Published:** 2025-05-05

**Authors:** Kanchana Yadav, Trishala Das, Andrew M. Lynn

**Affiliations:** School of Computational and Integrative Sciences, Jawaharlal Nehru University, New Delhi, 110067 India

**Keywords:** DNA damage repair genes, Immune stimulator genes, Immune inhibitor genes, MHC-related gene expression, Gene mutation, Cancer, Cancer, Computational biology and bioinformatics, Genetics, Immunology, Biomarkers

## Abstract

DNA damage is a key factor in many human disorders, including cancer, chronic inflammation, and early aging. Genes involved in DNA damage repair (DDR) not only maintain genomic stability but also contribute to immune responses by regulating the expression of antimicrobial peptides and ligands that activate immune receptors. Various components of the DNA damage response (DDR), including DNA damage sensors, transducer kinases, and effector proteins, are capable of activating diverse immunological signaling pathways. While DDR gene mutations are common in cancers, their effects on immune characteristics are not well understood. We investigated how mutations in DDR genes influence the expression of immune regulatory genes, including immune stimulators, inhibitors, and genes related to the major histocompatibility complex (MHC) pathway. Using gene expression data from The Cancer Genome Atlas (TCGA) and mutation data from cBioPortal, we analyzed 264 DDR-related genes and 66 immune regulatory genes. These genes were clustered and categorized using Metascape, an integrative bioinformatics tool that applies enrichment-based analysis to group functionally related genes into clusters. The clustered genes were further validated through a literature review and the GeneCards database. We scored the change in immune regulatory gene expression in response to DDR gene mutations to identify differentially expressed immune stimulators, inhibitors, and MHC-related genes. Our analysis revealed positive and negative correlations between DDR gene mutations and the expression of immune modulators. These findings could help guide future cancer treatments based on biomarkers and immunotherapy strategies.

**Supplementary Information:**

The online version contains supplementary material available at 10.1038/s41598-025-99965-y.

## Introduction

One essential cellular mechanism that is crucial for healing DNA damage is DNA damage repair, which also has a major impact on the immune system^1^. Cancer remains a significant global health challenge, with incidence and mortality rates continuing to rise. According to the latest Global Cancer Statistics, an estimated 20 million new cancer cases and 9.7 million cancer-related deaths occurred worldwide in 2022^2^. Lung cancer continued to be the most common cause of cancer-related mortality, accounting for an estimated 1.8 million deaths (18%). Colorectal (9.4%), liver (8.3%), stomach (7.7%), and female breast (6.9%) cancers were next in line. Survival rates have improved for some cancers due to advancements in early detection and targeted therapies^3^. Notably, the five-year relative survival rate for all cancers combined has increased from 49% in the mid-1970s to 69% for those diagnosed between 2013 and 2019^4,5^. The 5-year survival rate for pancreatic cancer is poor, ranging from 2 to 9%, and there is little variation across high, low, and middle-income nations. These statistics underscore the urgent need for continued cancer research to improve patient outcomes and develop more effective therapeutic strategies^6,7^.

The complex interaction between DNA damage repair (DDR) and the immune system is necessary to prevent microbial infections and regulate tumor growth of tumors^8^. Although mutations in DNA damage repair (DDR) genes are prevalent across various malignancies, their impact on the modulation and regulation of the immune response remains poorly understood. Cancer treatment has evolved significantly over the decades, transitioning from conventional therapies such as surgery, chemotherapy, and radiation to more advanced approaches, including targeted therapies and immunotherapies^9^. According to the NIH report “Cancer Treatments: Past, Present, and Future, 2024,” early cancer treatment strategies primarily focused on eradicating rapidly dividing cells, often leading to severe side effects due to their lack of specificity^10^. The advent of targeted therapies, such as tyrosine kinase inhibitors and monoclonal antibodies, has improved treatment precision by specifically targeting oncogenic pathways^11,12^. Furthermore, immunotherapies, including immune checkpoint inhibitors and CAR-T cell therapies, have revolutionized cancer care by enhancing the body’s immune response against tumors^13^. However, despite these advancements, treatment resistance and variability in patient response remain major challenges. Understanding the role of DNA Damage Response (DDR) mutations in cancer is crucial, as these mutations influence genomic stability, impact treatment efficacy, and serve as potential targets for novel therapeutic interventions^14^. Therefore the effects of DDR gene mutations on the expression and function of immune regulatory genes, which are involved in immune stimulation, inhibition, and major histocompatibility complex (MHC) pathways, need to be studied further.

Genes associated with the MHC pathway, as well as immune stimulators and inhibitors, play a critical role in regulating the immune response to cancer^15^. The MHC pathway is essential for antigen presentation, influencing T-cell activation and immune surveillance^16^. Immune stimulators, such as *CD28* and *ICOS*, enhance T-cell responses, whereas immune inhibitors, including *PD-1* and CTLA-4, contribute to immune evasion by tumors^17^. These genes can be activated to stimulate the immune system to target cancer cells or to inhibit the immune response to prevent excessive damage^18–20^. The crucial role of immune regulatory genes in cancer immunology has been underscored by recent advancements in cancer treatment,particularly the development of immune checkpoint inhibitors (ICIs) targeting *PD-1* and *CTLA-4*^21,22^. These inhibitors have revolutionized cancer therapy by restoring T-cell activity and enhancing anti-tumor immune responses^23^. Notably, emerging evidence suggests that mutations in DNA damage repair genes can modulate the tumor immune microenvironment, thereby influencing the efficacy of ICIs^24,25^. DDR-deficient tumors often exhibit higher tumor mutational burden (TMB) and increased neoantigen presentation, which can enhance the response to *PD-1/PD-L1* blockade therapies^26^. These findings emphasize the interplay between immune regulatory genes, DDR mutations, and immunotherapy outcomes, highlighting the potential for biomarker-driven patient stratification in cancer treatment.

Through modification of cytokine production and immune cell infiltration, mutations in DDR genes can have a significant effect on immune-related genes. These modifications can either strengthen immune cell function or encourage an immunosuppressive environment, depending on the particular mutation and the surrounding circumstances^27,28^. For example, homologous recombination repair is compromised by mutations in *BRCA1* and *BRCA2*, which results in cytosolic DNA buildup and cGAS–STING pathway activation^29–31^. This activation enhances the expression of proinflammatory cytokines such as type I interferons, strengthening immune surveillance. By increasing the expression of proinflammatory cytokines such as type I interferons, this route strengthens immune surveillance. However, these tumors might also express more PD-L1, which would suppress T cell activation and aid in immune evasion^32^. Research has indicated that the deactivation of *BRCA1*,* BRCA2*, or *FANCD2* leads to intrinsic inflammatory signaling within tumors, which includes the release of proinflammatory cytokines such as *TNF-α*,* CXCL10*, and *CCL5*, as well as the recruitment of immune cells^29,30^. For example, in tissue-specific contexts, the combined loss of *TP53* and *BRCA1* results in the development of cancer, with signatures that include increased expression of immune checkpoint genes such as *PD-1* and *CTLA4*, as well as increased numbers of Th2 cells, regulatory T cells (Tregs), central memory cells, and exhausted T cells^33–35^. Increased *PD-L1* expression has been linked to an improved immune response and increased susceptibility to immune checkpoint inhibitors in patients with breast and ovarian malignancies with *BRCA1/BRCA2* mutations^36,29^.

Similarly, mutations in mismatch repair (MMR) genes, such as *MLH1* and *MSH2*, lead to microsatellite instability (MSI), which promotes the generation of numerous immunogenic neoantigens. The increased neoantigen burden enhances MHC class I-mediated antigen presentation, making tumor cells more susceptible to cytotoxic T cell-mediated lysis^37,38^. High levels of microsatellite instability (MSI-high) are frequently observed in colorectal tumors harboring *MLH1* or *MSH2* mutations. This molecular phenotype is associated with increased tumor-infiltrating cytotoxic T lymphocytes and improved responsiveness to immune checkpoint blockade therapies, such as PD-1 inhibitors^39^.

*ATM* mutations can lower the production of MHC class I molecules, which can impair T-cell identification and diminish antigen presentation due to faulty DNA double-strand break repair. Furthermore, *ATM* depletion is linked to the increased expression of immune checkpoint molecules, such as *PD-L1*, which aids in immune evasion^40,41^.

*POLQ* mutations are involved in alternative end-joining repair, and *POLQ*-deficient tumors may present a greater mutational burden, which could improve the presentation of neoantigens and immune stimulation. *POLQ* mutations are associated with increased T-cell infiltration and mutational burden in breast cancer, indicating their dual involvement in immune response modulation^42,43^.

*ERCC1* mutations are associated with increased expression of immune checkpoint molecules and greater immune infiltration in non-small cell lung cancer (NSCLC), which affect how cancer responds to immunotherapy^44^. *ERCC1* deficient non-small cell lung cancer (NSCLC) cells display an amplified type *I IFN* transcriptomic signature and reduced ERCC1 expression is associated with increased lymphocytic infiltration in isogenic cell lines and samples^45^. Emerging evidence suggests that mutations in DNA damage repair (DDR) genes can modulate the expression of immune stimulatory and inhibitory molecules, as well as components of the MHC pathway. Nevertheless, the breadth and functional consequences of these associations remain underexplored. In this study, we systematically analyzed mutations in DDR genes across multiple functional categories and evaluated their impact on the expression of 66 immune stimulatory genes. These genes were stratified into 10 distinct functional groups based on their immunological roles, enabling a comprehensive assessment of the relationship between DDR gene alterations and immune signaling pathways.

The primary objective of this study was to elucidate the impact of mutations in DDR genes on the expression of immune stimulatory and inhibitory genes, as well as genes involved in the MHC pathway. This investigation was guided by two main aims. First, we sought to determine whether the effects of DDR gene mutations on immune-related gene expression vary across distinct DDR gene categories. Given that each DDR category is associated with unique biological, molecular, and cellular functions, we hypothesized that mutations within different DDR pathways may exert differential effects on immune gene regulation. Second, we systematically categorized 264 immune-related genes into 14 groups according to DDR pathway associations, and 66 immune-related genes into 10 functional groups based on their immunological roles. Using this classification framework, we evaluated whether mutations in specific DDR gene categories selectively modulate the expression of immune-related genes within particular pathways. This approach enables a nuanced understanding of how distinct DDR gene categories may differentially influence immune stimulators, immune inhibitors, and MHC-related genes.

These findings indicate that mutations in DDR genes can substantially influence the expression of immune stimulatory and inhibitory genes, as well as those involved in the MHC pathway. Elucidating the interplay between DDR gene alterations and immune regulatory pathways may have important implications for the development of novel cancer immunotherapies that target both genomic instability and immune modulation.

The studies selected for this analysis encompassed a broad spectrum of cancer types, making them well-suited for examining the relationship between DDR gene mutations and immune gene expression across diverse tumor contexts. The inclusion of large and heterogeneous sample sets enhanced both the statistical power and the generalizability of the findings. In total, 10,967 samples were analyzed, providing a robust dataset that enabled the identification of statistically significant associations and offered valuable insights into the underlying biological mechanisms. Moreover, the studies incorporated in this work were published in high-impact, peer-reviewed journals and featured well-curated datasets, thereby ensuring the reliability and accuracy of the analytical outcomes.

## Materials and methods

In this study, we utilized a comprehensive dataset comprising 32 distinct human cancer types from The Cancer Genome Atlas (TCGA) PanCancer Atlas to investigate the impact of DNA damage repair (DDR) gene mutations on the expression of immune-related genes. The datasets were obtained from the cBioPortal for Cancer Genomics (www.cbioportal.org), specifically from the TCGA PanCancer studies, and encompass a diverse range of malignancies, including cancers of the adrenal gland, biliary tract, bladder/urinary tract, bowel, breast, central nervous system (CNS), cervix, esophagus/stomach, eye, head and neck, kidney, liver, lung, lymphoid tissue, myeloid lineage, ovary/fallopian tube, pancreas, pleura, prostate, skin, soft tissue, testis, thymus, thyroid, uterus, and uterine carcinosarcoma.

This broad representation of cancer types was selected to ensure diversity and to provide a robust foundation for statistical analyses and correlation studies. Such a comprehensive approach enabled a detailed examination of the relationship between DDR gene mutations and the expression of immune stimulators, immune inhibitors, and MHC pathway-related genes across a pan-cancer context. The number of samples analyzed for each cancer type is detailed in Supplementary Ttable S1.

In this analysis, we employed mRNA expression Z-score data from the TCGA PanCancer Atlas (blca_tcga_pan_can_atlas_2018) for 66 immune-related genes, encompassing immune stimulators, immune inhibitors, and genes associated with the major histocompatibility complex (MHC) pathway. Z-scores were calculated from log-transformed RNA-Seq V2 RSEM data, facilitating the assessment of relative gene expression levels in tumor tissues compared to normal controls. Positive Z-scores indicate gene upregulation in tumor samples, while negative Z-scores denote downregulation.

As no single resource comprehensively listed all DNA damage repair (DDR) genes, we curated a set of 264 unique DDR genes by integrating information from multiple peer-reviewed studies and publicly available databases^46–52^. Similarly, we compiled the list of immune-related genes used in this study from published literature, resulting in 45 immune stimulator genes, 17 immune inhibitor genes, and 4 MHC pathway-related genes^53,54^.

To classify 264 DNA damage repair (DDR) genes into 14 functional categories and immune-related genes into 10 categories, we utilized Metascape (RRID: SCR_014687), a comprehensive gene annotation and analysis platform. Metascape integrates multiple biological databases, including Gene Ontology (GO) and the Kyoto Encyclopedia of Genes and Genomes (KEGG; RRID: SCR_012773)^55,56^, to perform functional enrichment analyses and similarity-based clustering of gene sets^57^. Gene clustering within Metascape leverages the Molecular Complex Detection (MCODE) algorithm (RRID: SCR_015828), which identifies highly interconnected regions in protein–protein interaction (PPI) networks, thereby revealing potential functional modules or protein complexes. The MCODE algorithm employs a seed-and-extend strategy, wherein highly connected ‘seed’ nodes are first detected and subsequently expanded to include neighboring nodes with strong connectivity, enabling robust module construction. Additionally, data preprocessing, statistical analyses, and visualizations were performed using Python-based libraries, including Pandas, NumPy (RRID: SCR_008633), Seaborn, and Matplotlib (Pyplot; RRID: SCR_008624). These tools collectively facilitated the comprehensive categorization and visualization of gene functions across the datasets.

### Classification of 264 DNA damage repair genes into 14 categories on the basis of their repair mechanism

Recent advances in gene annotation and pathway analysis have significantly improved our ability to decipher the functional roles and underlying mechanisms of genes involved in complex biological processes. In this study, we curated a comprehensive list of 264 DNA damage repair (DDR) genes from peer-reviewed publications and established databases. To functionally categorize these genes according to their respective repair mechanisms, we employed the Metascape platform (RRID: SCR_014687) for gene annotation, enrichment analysis, and functional clustering. The classification output was further validated through cross-referencing with published literature and the GeneCards database (www.genecards.org; RRID: SCR_002773)^58–60^.

Based on this integrative analysis, the 264 DDR genes were stratified into 14 distinct functional categories: base excision repair (BER), direct damage reversal, mismatch excision repair (MMR), nucleotide excision repair (NER), homologous recombination, Fanconi anemia pathway, repair of DNA crosslinks and other DNA adducts, nonhomologous end joining (NHEJ), modulation of nucleotide pools, DNA polymerases, nucleases involved in editing and processing, ubiquitination and other post-translational modifications, structural modification, cell cycle checkpoints, and a subset of genes with known or putative roles in DNA repair. This categorization provides a detailed framework for understanding the functional landscape of DDR genes and their contributions to genome integrity.

### Classification of immune-related genes based on their specific functions

From the literature review, we obtained the names of 66 immune-related genes, which were classified as immune stimulators, inhibitors, and MHC pathway-related genes. A total of 45 genes functioned as immune stimulator genes, 17 as immune inhibitor genes, and four as MHC pathway-related genes. To categorize these genes according to their specific functions, we performed gene annotation, enrichment analysis, and functional clustering classification using MetaScape (RRID: SCR_014687)^60–63^. The resulting ten categories were validated using published papers and the Gene Card database. These categories include receptors, co-inhibitors, ligands, costimulators, antigen presentation, cell adhesion, immune suppression, T cell activation, anti-inflammatory cytokines, and Th0 to Th2 differentiation.

### Correlation study between DNA damage repair gene mutations and expression patterns in immune stimulators, immune inhibitors, and MHC pathway-related genes

We obtained mutation data for DNA damage repair (DDR) genes and expression data for immune-related genes from the TCGA Pan-Cancer Atlas dataset via cBioPortal. Only samples with matching sample and patient identifiers were retained for downstream analysis. DDR gene samples were subsequently stratified into two groups based on mutation status: mutated and non-mutated. We then merged and mapped the sample identifiers of both groups with those corresponding to immune-related genes, specifically immune stimulators, immune inhibitors, and genes involved in the major histocompatibility complex (MHC) pathway. This integrative analysis enabled us to compare the expression profiles of immune stimulators, inhibitors, and MHC pathway-associated genes between the mutated and non-mutated DDR gene groups, thereby facilitating the investigation of potential associations between DDR mutations and immune gene regulation across cancer types.

## Results

### Classification of DNA damage repair genes into different categories

The results of the clustering and gene annotation analyses suggested that the 264 genes under study fell into multiple distinct DNA damage repair pathway clusters. After applying the clustering algorithm in Metascape, we classified the genes into 14 categories. Metascape employs an enrichment-based clustering approach that integrates multiple functional annotation sources, such as Gene Ontology (GO) and KEGG pathways, to group genes based on shared biological functions and pathway involvement. This method ensures that genes with similar biological roles are systematically categorized, enhancing the interpretability of functional analyses. To validate our clustering results, we compared our gene clusters with those from published studies and the GeneCards database. The validation process involved a thorough literature search to identify relevant studies reporting gene functions or interactions. The validation results confirmed that the genes in each of the 14 clusters exhibited similar biological functions or interactions, further supporting the robustness of our clustering and categorization approach. The validation results are provided in the supplementary data of Tables S2–S15.

**Fig. 1:**
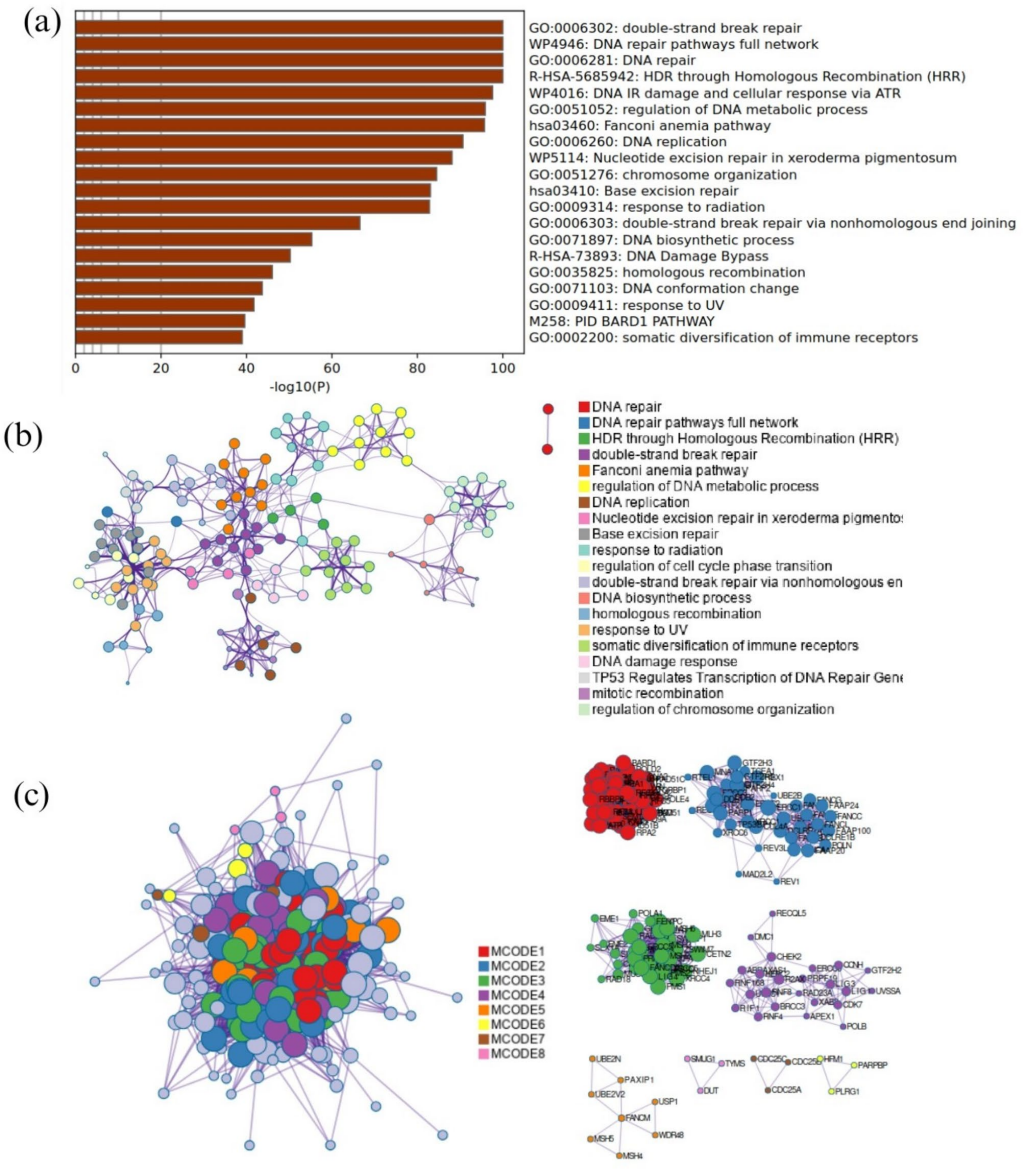
Comprehensive visualization of enriched terms and protein–protein interaction (PPI) networks derived from 264 DNA damage repair (DDR) genes. (**a**) Bar graph showing the top nonredundant enrichment clusters; the y-axis lists significant Gene Ontology (GO) terms and pathways, while the x-axis represents the –log10(p) values indicating statistical significance. (**b**) Network view of enriched terms, with nodes colored by cluster ID to illustrate intra- and inter-cluster similarities. Nodes represent biological processes or pathways, with closely connected nodes sharing common genes and indicating functional relatedness. (**c**) Protein–protein interaction network constructed from the 264 DDR genes, including the Molecular Complex Detection (MCODE) components. The large network cluster shows proteins (nodes) and their interactions (edges), with color-coded nodes representing different MCODE components. Smaller subnetworks on the right display individual MCODE modules, labeled and color-coded to match the main network, with a legend provided for clarity.

**Fig. 2:**
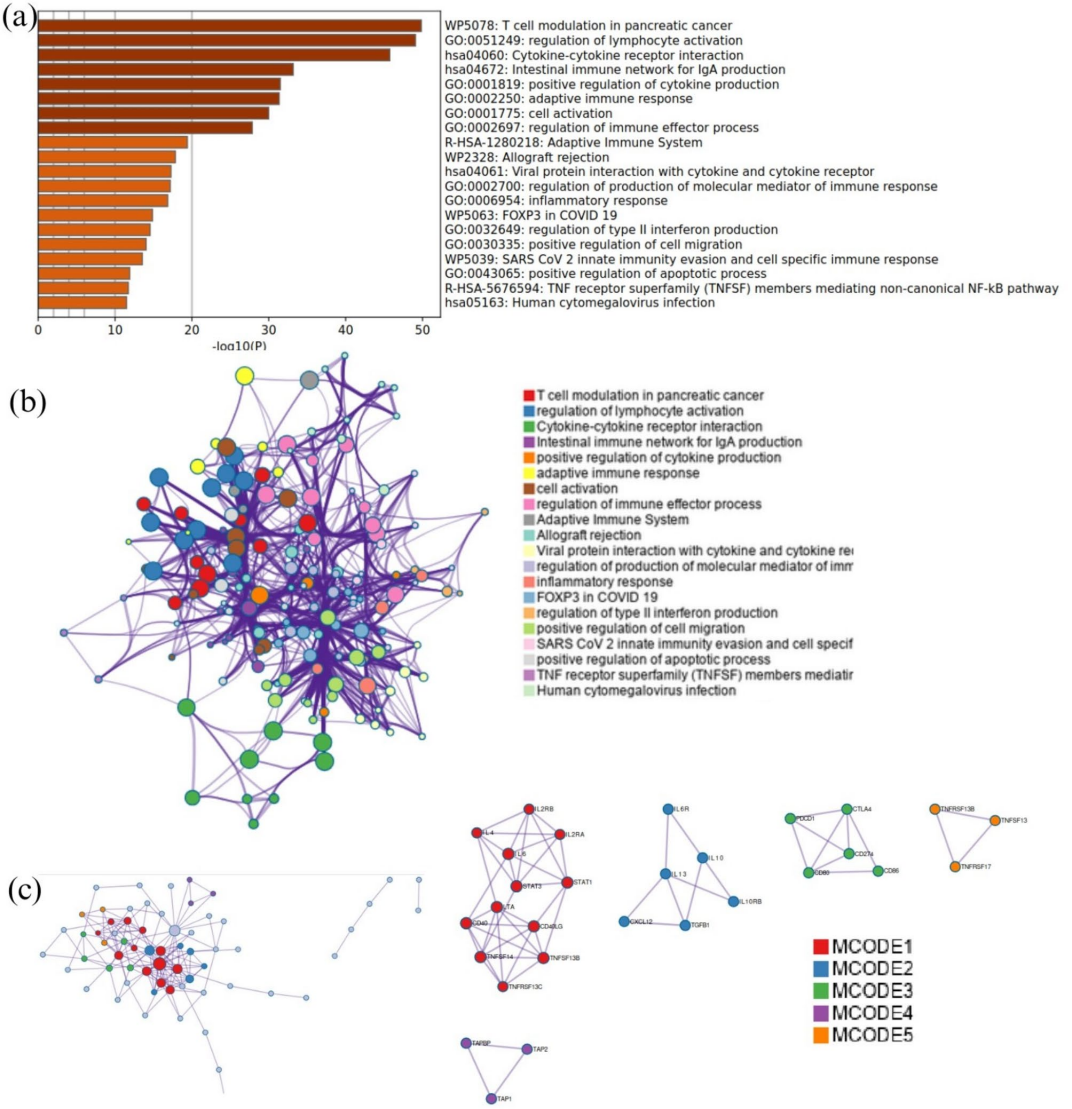
Visualization of enriched terms and protein–protein interaction (PPI) networks derived from 66 immune stimulators, inhibitors, and MHC pathway-related genes. (**a**) Bar graph of the top nonredundant enrichment clusters. The y-axis lists significant Gene Ontology (GO) terms and pathways, and the x-axis represents –log10(p) values, indicating statistical significance. (**b**) Network of enriched terms, with nodes colored by cluster ID, indicating intra- and inter-cluster similarities. Nodes represent specific biological processes, and edges indicate relationships or functional similarities. (**c**) Protein–protein interaction network constructed from the 66 immune-related genes, highlighting Molecular Complex Detection (MCODE) components. The main network shows interconnected nodes (proteins) and edges (interactions), with nodes color-coded by MCODE components. Smaller subnetworks on the right present individual MCODE modules, labeled and color-matched to their counterparts in the larger network. A color legend is provided at the bottom right for reference.

**Fig. 3:**
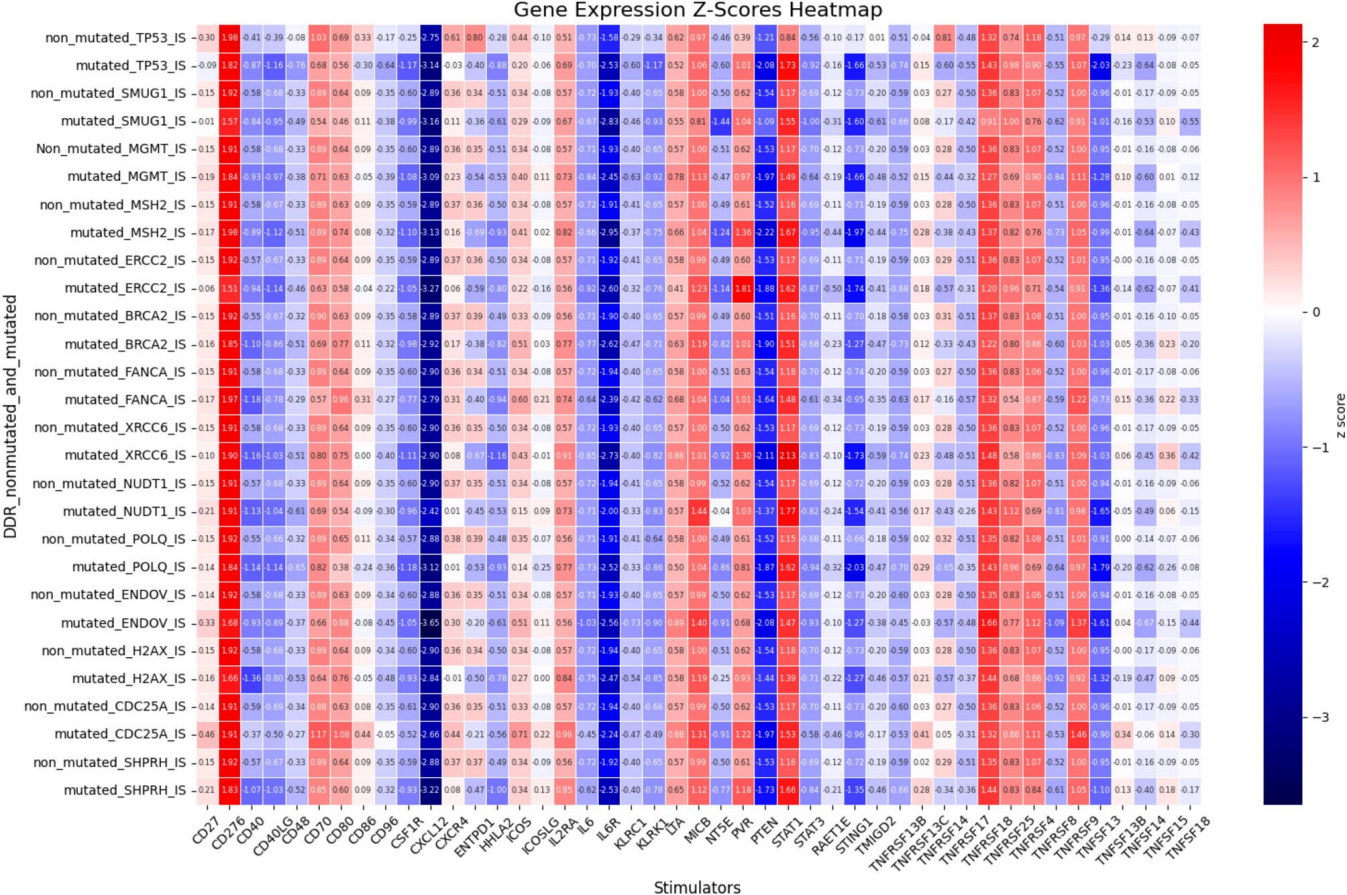
Expression changes in immune stimulator genes in relation to mutations in DNA damage repair (DDR) genes. The x-axis indicates immune stimulator genes, and the y-axis lists DDR genes including *TP53*,* SMUG1*,* MGMT*,* MSH2*,* ERCC2*,* BRCA2*,* FANCA*,* XRCC6*,* NUDT1*,* POLQ*,* ENDOV*,* H2AX*,* SHPRH*, and *CDC25A*. Each row presents immune stimulator gene expression under two conditions: non-mutated and mutated DDR gene status. Expression values are shown as Z-scores, representing the log2 fold change in mean expression between the two conditions.

**Fig. 4:**
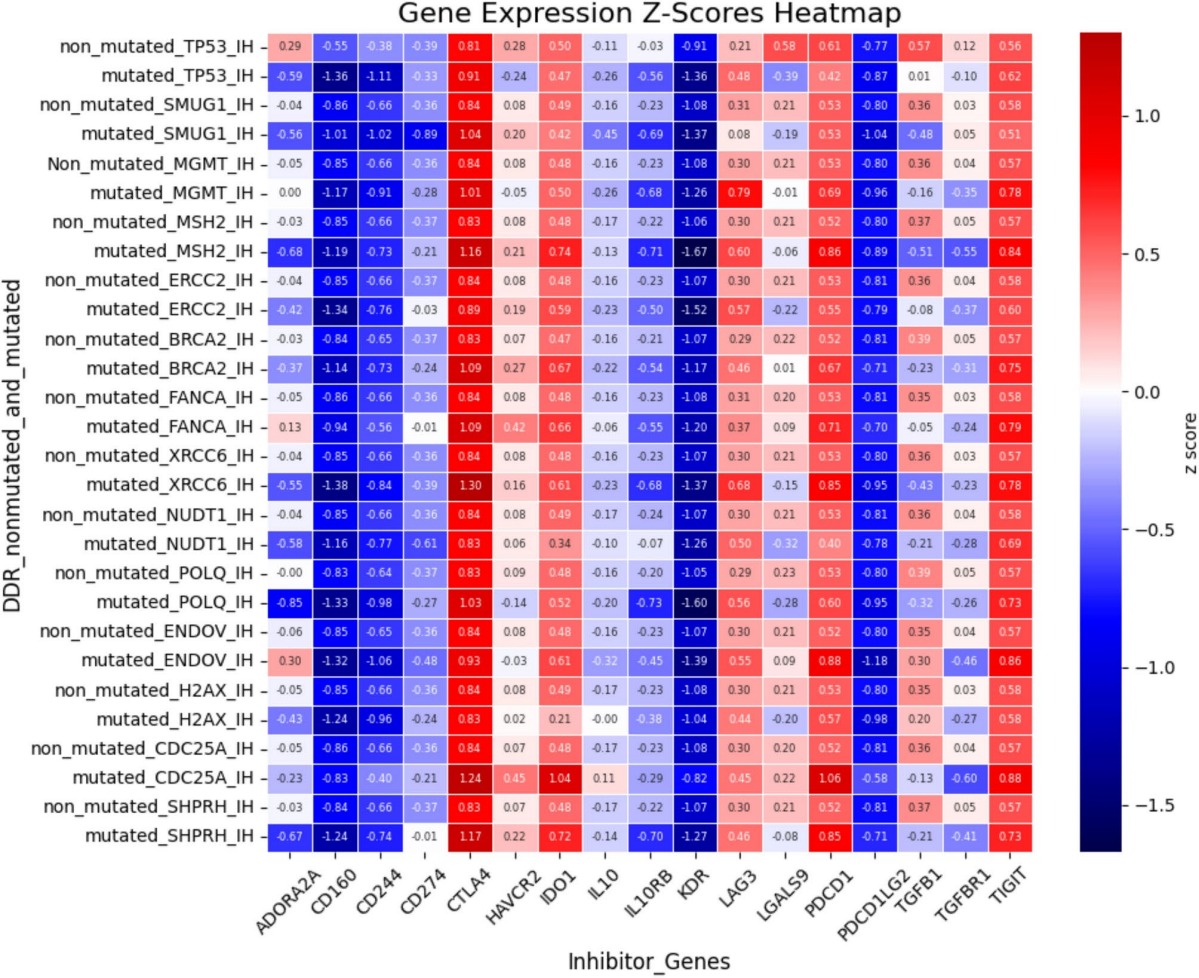
Expression changes in immune inhibitor genes associated with mutations in DNA damage repair (DDR) genes. The x-axis represents immune inhibitor gene names, and the y-axis lists DDR genes including *TP53*,* SMUG1*,* MGMT*,* MSH2*,* ERCC2*,* BRCA2*,* FANCA*,* XRCC6*,* NUDT1*,* POLQ*,* ENDOV*,* H2AX*,* SHPRH*, and *CDC25A*. Each row compares immune inhibitor gene expression under two conditions: non-mutated and mutated DDR gene status. Expression values are presented as Z-scores, representing the log2 fold change in mean expression between the two conditions.

**Fig. 5:**
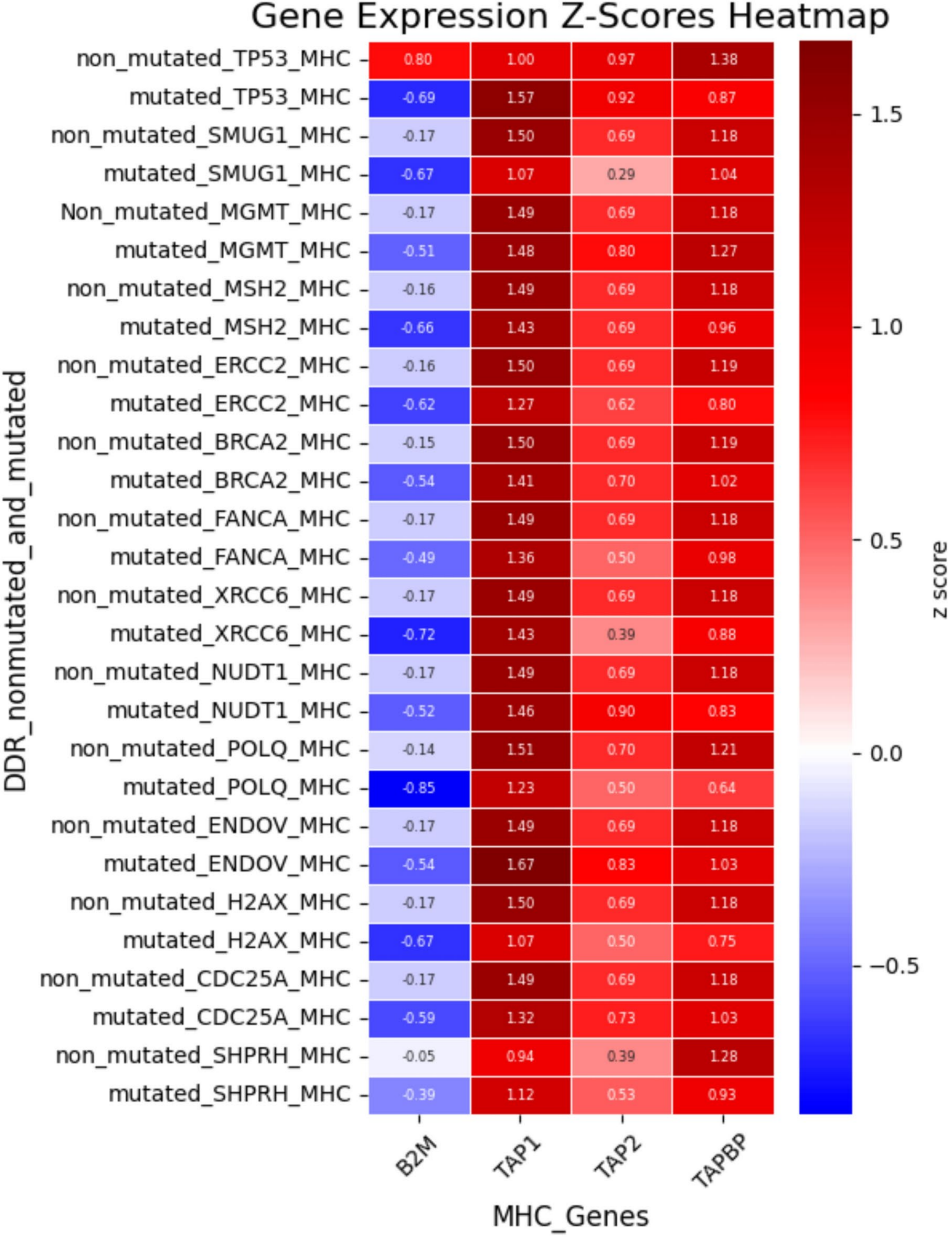
Expression changes in MHC pathway-related genes associated with mutations in DNA damage repair (DDR) genes. The x-axis shows MHC pathway-related gene names, and the y-axis lists DDR genes including *TP53*,* SMUG1*,* MGMT*,* MSH2*,* ERCC2*,* BRCA2*,* FANCA*,* XRCC6*,* NUDT1*,* POLQ*,* ENDOV*,* H2AX*,* SHPRH*, and *CDC25A*. Each row presents the expression of MHC pathway genes under two conditions: non-mutated and mutated DDR gene status. Expression levels are represented as Z-scores, indicating the log2 fold change in mean expression between the two conditions.

The clustering and functional classification of the 264 DNA damage repair (DDR) genes into 14 distinct categories are presented in Figure as Fig. 1; Table 1. To investigate the impact of DDR mutations on immune-related gene expression, we randomly selected one representative gene from each DDR category Fig. 2. For each selected gene, we assessed changes in the expression of immune-related genes, specifically those involved in immune stimulation, inhibition, and MHC pathways—when the DDR gene was mutated. The results of this analysis are visualized as heatmaps in Figures as Figs. 3, 4 and 5 and detailed in Supplementary Table S17. Representative gene selection was conducted using a random sampling strategy to minimize selection bias and ensure equitable representation across functional categories. Future analyses will extend this approach to examine the effects of mutations across all 264 DDR genes on immune gene expression profiles.

This analysis is crucial for understanding the organization of proteins in biological systems, identifying key protein complexes, and revealing functional modules within the overall network. By focusing on the most interconnected and potentially relevant subnetworks, this analysis provides insights into the critical proteins and complexes that may be important for further biological or therapeutic research.

**Table 1:** Shows the classification of 264 DNA damage repair genes into 14 categories.

SN	Repair pathway	Genes name
1	Base excision repair (BER)	*UNG*,* SMUG1*,* MBD4*,* TDG*,* OGG1*,* MUTYH*,* NTHL1*,* MPG*,* NEIL1*,* NEIL2*,* NEIL3*,* APEX1*,* APEX2*,* PNKP*,* PARP2*,* PARG*
2	Direct reversal of damage	*MGMT*,* ALKBH2*,* ALKBH3*,* ASCC3*
3	Mismatch excision repair (MMR)	*MSH2*,* MSH3*,* MSH6*,* MLH1*,* PMS2*,* MSH4*,* MSH5*,* MLH3*,* PMS1*,* HMGB1*,* PMS2P3*
4	Nucleotide excision repair (NER)	*XPC*,* RAD23B*,* CETN2*,* RAD23A*,* XPA*,* DDB1*,* DDB2*,* RPA1*,* RPA2*,* RPA3*,* ERCC2*,* GTF2H1*,* GTF2H2*,* GTF2H3*,* GTF2H4*,* GTF2H5*,* ERCC5*,* ERCC1*,* ERCC4*,* ERCC8*,* ERCC6*,* UVSSA*,* XAB2*,* INO80*,* ERCC3*,* TCEA1*
5	Homologous recombination	*RAD51*,* RAD51B*,* RAD51D*,* HELQ*,* SWI5*,* SWSAP1*,* ZSWIM7*,* SPIDR*,* DMC1*,* XRCC2*,* XRCC3*,* RAD52*,* RAD54L*,* RAD54B*,* BRCA1*,* BARD1*,* SMC5*,* SMC6*,* SEM1*,* RAD50*,* NBN*,* RBBP8*,* MUS81*,* EME1*,* EME2*,* SLX1A*,* SLX1B*,* GEN1*,* PPP4C*,* PPP4R2*,* RTEL1*,* BLM*,* RMI1*,* TOP3A*,* WRN*,* RECQL4*,* ATM*,* NABP2*,* MORF4L1*,* RMI2*,* PARPBP*,* BRCA2*,* BRIP1*,* PALB2*,* RAD51C*,* SLX4*
6	Fanconi anemia- Tolerance and repair of DNA crosslinks and other adducts in DNA	*FANCA*,* FANCB*,* FANCC*,* FANCD2*,* FANCE*,* FANCF*,* FANCG*,* FANCI*,,* FANCL*,* FANCM*,* FAAP20*,* FAAP24*,* FAAP100*,* UBE2T*,* FAN1*
7	Nonhomologous end-joining	*XRCC6*,* XRCC5*,* PRKDC*,* LIG4*,* XRCC4*,* DCLRE1C*,* NHEJ1*,* APLF*,* HMCES*,* PARP3*,* RNF168*,* TDP1*,* TDP2*,* DNTT*,* POLM*,* DCLRE1B*,* DCLRE1A*,* HMGB2*
8	Modulation of nucleotide pools	*NUDT1*,* DUT*,* RRM2B*,* DNPH1*,* NUDT15*,* NUDT18*
9	DNA polymerases	*POLA1*,* POLB*,* POLD1*,* POLD2*,* POLD3*,* POLD4*,* POLE*,* POLE2*,* POLE3*,* POLE4*,* REV3L*,* MAD2L2*,* REV1*,* POLG*,* POLH*,* POLI*,* POLQ*,* POLK*,* POLL*,* POLN*,* PRIMPOL*
10	Editing and processing nucleases	*FEN1*,* TREX1*,* TREX2*,* EXO1*,* APTX*,* ENDOV*,* DNA2*,* EXO5*,* BCAS2*,* PLRG1*,* TYMS*,* NMNAT1*,* SPO11*
11	Chromatin structure and modification	*H2AX*,* CHAF1A*,* SETMAR*,* ATRX*,* SMARCA4*,* SMARCAD1*,* SMARCC1*,* SOX4*,* PER1*
12	Ubiquitination and modification	*UBE2A*,* UBE2B*,* SHPRH*,* HLTF*,* RNF8*,* RNF4*,* UBE2V2*,* UBE2N*,* USP1*,* WDR48*,* RAD18*,* HERC2*,* RBX1*
13	Other identified genes with known or suspected DNA repair function	*RECQL*,* RECQL5*,* IDH1*,* PARP4*,* PPP4R1*,* MMS19*,* HFM1*,* PDS5B*,* PAXIP1*,* SHLD1*,* SHLD2*,* MRE11A*,* LIG3*,* XRCC1*,* PARP1*,* RFC1*,* RFC2*,* RFC3*,* RFC4*,* RFC5*,* LIG1*,* PRPF19*,* PCNA*,* RAD9A*,* KAT5*,* UIMC1*,* SPRTN*,* PARK7*,* TOPBP1*,* TP53*,* TP53BP1*,* RIF1*,* CLK2*,* AEN*,* ALKBH1*,* GADD45A*,* PTEN*
14	Cell cycle checkpoints	*CDC25A*,* CDC25B*,* CDC25C*,* CDC5L*,* CUL3*,* CUL5*,* H2AFX*,* MNAT1*,* PLK3*,* RAD9B*,* WEE1*,* MDC1*,* CHEK2*,* ABRAXAS1*,* BRCC3*,* CUL4A*,* CDK7*,* CCNH*,* RPA4*,* TELO2*,* GADD45G*,* ATR*,* ATRIP*,* RAD1*,* RAD17*,* CHEK1*,* MPLKIP*,* TTK*

Table one presents a comprehensive list of genes involved in 14 distinct DNA repair pathways. A total of 264 DNA damage repair genes have been categorized into these pathways based on their respective repair mechanisms. Column 1 displays the serial number, Column 2 lists the repair pathways, and Column 3 provides the corresponding genes associated with each pathway.

### Classification of immune-related genes into different categories

The clustering function of Metascape (RRID: SCR_014687) is based on the MCODE algorithm. The same algorithm was applied to classify the immune-related genes into 10 categories. The 66 examined genes belonged to several groups. After applying the clustering technique, we identified immune-related genes that fell into many separate clusters according to their unique roles. Genes involved in immunity that are grouped together have similar biological functions. We divided the 66 immune-related genes into ten categories based on their functional similarities and dissimilarities. To corroborate our clustering findings, we validated our gene clusters using published articles and gene card databases that have previously examined the roles of these genes. The validation results are shown in Supplementary Table S16. The validation procedure included a thorough literature search to identify pertinent papers that discussed the connections or functions of genes. The robustness of our clustering and categorization approach was further demonstrated by our confirmation that the genes in each of the 10 clusters displayed similar biological roles or interactions, based on the validation results. The results of clustering and classification of the 66 immune-related genes into 10 categories are shown in Figure as Fig. 2; Table 2.

**Table 2:** Shows the classification of 66 immune-related genes into 10 categories.

SN	Genes function	Genes name
1	Receptor	*CD27 (TNFRSF7)*,* CD40*,* CTLA4*,* HAVCR2 (TIM-3)*,* ICOS*,* IL2RA (CD122)*,* PDCD1 (PD-1)*,* TIGIT*,* TNFRSF14 (HVEM)*,* TNFRSF18 (CD357*,* GITR)*,* TNFRSF4 (CD134*,* OX40)*,* TNFRSF9 (CD137*,* 4-1BB)*,* CD48*,* KLRC1*,* TGFBR1*,* CSF1R*,* CXCR4*,* IL6R*,* TNFRSF13C*,* ADORA2A*,* CD244*
2	Coinhibitor	*CD274 (PD-L1)*,* CD276 (B7-H3)*,* PDCD1LG2 (PD-L2*,* CD273*,* B7-DC)*,
3	Ligand	*CD40LG*,* CD70*,* TGFB1*,* STAT3*,* LGALS9*,* LTA*,* CXCL12*,* TNFSF13*
4	Costimulator	*CD80 (B7-1)*,* ICOSLG (B7-H2)*,* CD86*,* PTEN*,* STAT1*,* STING1*,* TNFRSF13B*,* TNFRSF17*,* TNFRSF25*,* TNFRSF8*,* TNFSF13B*,* TNFSF14*,* TNFSF15*,* TNFSF18*
5	Antigen presentation (MHC Class I and MHC Class II)	*LAG3*,* B2M*,* TAP1*,* TAPBP*,* RAET1E*,* TAP2*,* MICB*
6	Cell adhesion	*CD96*,* NT5E*,* PVR*,* ENTPD1*
7	Immune suppression	*CD160*,* IDO1*,* IL10RB*,* KDR*
8	T-cell activation	*HHLA2*,* KLRK1*
9	Anti-inflammatory cytokine	*IL10*
10	Differentiation of Th0 to Th2	*TMIGD2*,* IL6*

Table 2 presents the classification of 66 immune-related genes into 10 distinct pathways based on their specific functions. Column 1 displays the serial number, Column 2 describes the specific function of each immune-related gene, and Column 3 lists the genes associated with each biological pathway.

### Correlations between DNA damage repair gene mutations and immune regulatory gene expression across cancers

In this analysis, a simple threshold approach was used to determine whether the change in Z-scores between non-mutated and mutated samples was significant. The specific value (e.g., 0.5) was chosen as the cutoff. If the absolute difference in the Z scores of the immune-related genes in non-mutated and mutated DDR genes exceeded this threshold, the change was flagged as significant; otherwise, it was flagged as insignificant.

The simple threshold method using a Z-score difference of 0.5 plays a crucial role in our study by offering a straightforward yet effective approach to identify significant changes in gene expression between non-mutated and mutated samples. By setting this cut-off, we were able to systematically flag genes with notable differences in expression, ensuring that only biologically relevant variations were captured. This approach minimizes data noise and focuses on identifying key genes affected by DDR mutations, particularly those involved in immune functions. The consistent application of this threshold facilitates the reliable detection of differential expression, supporting meaningful comparisons and conclusions that align with existing literature. This method is essential for identifying critical targets within these biological pathways and has potential implications for therapeutic research.

In our study, we observed differential expression of various immunostimulatory genes that are known to affect DNA repair functions in the context of *TP53* gene mutations. The immunostimulatory genes that were differentially expressed when *TP53* was mutated included *CD40LG* and *TNFSF13*, which belong to the ligand group; *CD48*, *CSF1R*, *CXCR4*, *IL6R*, and *TNFRSF14*, which belong to the receptor group; and *CD86*, *PTEN*, *TNFSF14*, and *STING1*, which are costimulators. *ENTPD1* and *PVR* are associated with cell adhesion, *HHLA2* and *KLRK1* are involved in T-cell activation, and *TMIGD2* is linked to the differentiation of Th0 to Th2 cells. The differentially expressed immune inhibitory genes included *ADORA2A*, *CD244*, and *HAVCR2*, which are receptors; *CD160* and *IL10RB*, which are involved in immune suppression; and *LGALS9* and *TGFB1*, which belong to the ligand group. MHC pathway-related genes, whose expression changes when the *TP53* gene is mutated, include *B2M* and *TAPBP*, which are involved in antigen presentation.

In our study, we observed that mutations in *SMUG1*, a key component of the base excision repair (BER) pathway, were associated with significant changes in the expression of several immunostimulatory genes. Specifically, when *SMUG1* is mutated, differentially expressed immune stimulatory genes include *IL6R*, which belongs to the receptor group; *NT5E* and *ENTPD1*, which are associated with cell adhesion; and *STING1*, which belongs to the costimulator category. Additionally, differentially expressed immune inhibitory genes in the presence of SMUG1 mutations include *ADORA2A*, a receptor; *CD274*, an inhibitor; and *TGFB1*, a ligand. No MHC genes were differentially expressed when SMUG1 was mutated.

Mutations in the *MGMT* gene, which plays a crucial role in the direct reversal of DNA damage, are linked to significant alterations in the expression of various immune stimulator genes. Notably, *MGMT* mutations resulted in differential expression of *ENTPD1*, which is involved in cell adhesion; *IL6R* and *TNFRSF14*, both of which are receptor-related; and *STING1*, which is part of the costimulator group. Additionally, the immune inhibitory gene *TGFB1*, which belongs to the ligand group, was differentially expressed when *MGMT* was mutated. However, no genes associated with the MHC pathway were mutated in conjunction with *MGMT* mutations.

Mutations in *MSH2*, a crucial component of the mismatch repair (MMR) pathway, significantly affect the expression of various immunostimulatory genes. Alterations in *MSH2* led to changes in the expression of genes such as *CSF1R*, *IL6R*, and *TNFRSF14* (receptor group); *ENTPD*, *NT5E*, and *PVR* (cell adhesion group); and *PTEN*, *STAT1*, and *STING1* (costimulatory group). Furthermore, mutations in *MSH2* resulted in differential expression of immune inhibitory genes, including *ADORA2A* and *TGFBR1* (receptor family), *KDR* (immune suppression group), and *TGFB1* (ligand group). However, none of the genes associated with the MHC pathway were differentially expressed when *MSH2* was mutated.

Mutation of *ERCC2*, a gene involved in the nucleotide excision repair pathway, leads to the differential expression of various immunostimulatory genes. Specifically, *ENTPD1*, *PVR*, *NT5E* (cell adhesion group), *IL6R*, *TNFRSF14* (receptor group), and *STING1* (costimulator group) showed altered expression. However, no changes in the expression of immune inhibitory genes or genes related to the MHC pathway were detected when *ERCC2* was mutated.

*BRCA2*, a gene involved in homologous recombination, showed specific changes in expression when mutated. Mutation of the *BRCA2* DNA damage repair (DDR) gene leads to differential expression of several immunostimulatory genes, including *CD40*, *IL6R*, *TNFRSF14* (receptor group), *ENTPD1* (cell adhesion group), and *STING1* (costimulator group). Additionally, the immune inhibitory gene *TGFB1* (ligand category) was differentially expressed when *BRCA2* was mutated. However, no genes associated with the MHC pathway were differentially expressed in response to *BRCA2* mutations.

*FANCA*, a gene involved in the Fanconi anemia pathway, responsible for the tolerance and repair of DNA crosslinks and other adducts, exhibits specific changes in gene expression when mutated. Mutation of the *FANCA* gene leads to the differential expression of immunostimulatory genes, including *CD40* (receptor category), *NT5E*, and *ENTPD* (cell adhesion category). However, no immune inhibitory genes or genes related to the MHC pathway were differentially expressed when *FANCA* was mutated.

*XRCC6*, a gene involved in the nonhomologous end joining (NHEJ) pathway, shows significant changes in gene expression when mutated. Mutations in *XRCC6* lead to the differential expression of several immune stimulatory genes, including *CD40*, *CSF1R*, *IL6R*, *TNFRSF14* (receptor category), *ENTPD1* (cell adhesion), *HHLA2* (T-cell activation category), and *STAT1* (costimulator category). Additionally, the mutation affected immune inhibitor genes, with *ADORA2A* (receptor), *CD160* (immune suppression), and *TGFB1* (ligand) exhibiting differential expression. The MHC pathway-related gene, *B2M*,* was* also differentially expressed when *XRCC6* was mutated.

*NUDT1*, a gene involved in the modulation of nucleotide pools within the DNA damage response (DDR) category, shows specific gene expression changes when mutated. Mutations in *NUDT1* result in the differential expression of several immune stimulatory genes, including *CD40*, *TNFRSF14*, *TNFSF13* (receptor category), *ENTPD1* (cell adhesion), and *STAT1* and *STING1* (costimulator category). Additionally, the mutation affected immune inhibitor genes, with *ADORA2A* (receptor), *LGALS9* and *TGFB1* (ligand category) showing differential expressions. However, none of the genes associated with the MHC pathway were mutated in conjunction with *NUDT1* mutations.

*POLQ*, a gene belonging to the DNA polymerase family, exhibits notable changes in expression when mutated. Mutations in *POLQ* result in differential expression of several immunostimulatory genes, including *CD40*, *CSF1R*, *IL6R*, *TNFRSF14*, *TNFSF13* (receptor family), *STING1* (costimulator category), and *ENTPD1* (immune system). Additionally, the mutation led to the differential expression of immune inhibitor genes such as *ADORA2A* (receptor family), *IL10RB*, *KDR* (immune suppression), *LGALS9* and *TGFB1* (ligand category). Moreover, MHC pathway-related genes, B2M and *TAPBP*, which are involved in antigen presentation, were also differentially expressed when *POLQ* was mutated.

*ENDOV*, a gene involved in editing and processing nucleases, exhibits distinct changes in expression when mutated. Mutations in *ENDOV* lead to the differential expression of several immunostimulatory genes, including *CXCL12* and *TNFSF13* (ligand category), *ENTPD1* (immune inhibitor), *IL6R* and *TNFRSF14* (receptor category), and *PTEN*, *TNFRSF8*, *TNFSF14*, and *STING1* (costimulator category). However, no immune inhibitor- or MHC pathway-related genes were differentially expressed when *ENDOV* was mutated.

*H2AX*, a gene associated with chromatin structure and modification, exhibits notable changes in its expression when mutated. Specifically, mutations in *H2AX* lead to the differential expression of immune stimulatory genes, including *CD40*, *IL6R*, *TNFRSF14* (receptor category), and *STING1* (costimulator group). Additionally, when *H2AX* was mutated, no immune inhibitor genes were found to be differentially expressed. However, this mutation results in the differential expression of the MHC pathway-related gene *B2M*.

Mutation of *CDC25A*, a gene involved in cell cycle checkpoints, results in distinct changes in gene expression. Mutation of the *CDC25A* DDR gene leads to the differential expression of the immunostimulatory gene *ENTPD1*, which is associated with cell adhesion. Additionally, several immune inhibitory genes, including *DO1* (immune suppression), *PDCD*, *TGFBR1*, *ADORA2A* (receptor category), and *TGFB1* (ligand category), were differentially expressed. However, no MHC pathway-related genes were differentially expressed when *CDC25A* was mutated.

*SHPRH*, a gene involved in ubiquitination and modification, exhibited significant changes in expression when mutated. Mutations in the *SHPRH* gene lead to the differential expression of several immune stimulatory genes, including *ENTPD1* and *PVR* (cell adhesion category), *HHLA2* (T-cell activation), *IL6R* and *TNFRSF14* (receptor category), and *STAT1* and *STING1* (costimulator category). However, no immune-inhibitory or MHC pathway-related genes were differentially expressed when *SHPRH* was mutated.

In our analysis of immune stimulator genes, we observed that when DNA Damage Repair (DDR) genes were mutated, *PVR* and *STAT1* consistently showed a positive correlation with increased expression levels. In contrast, the expression of other immune stimulator genes, including *CD40LG*, *CD48*, *CD86*, *CSF1R*, *CXCR4*, *ENTPD1*, *HHLA2*, *IL6R*, *KLRK1*, *PTEN*, *STING1*, *TMIGD2*, *TNFRSF14*, *TNFSF13*, and *TNFSF14*, was negatively correlated with DDR gene mutations. These findings underscore the intricate relationship between DNA damage repair (DDR) gene mutations and the regulation of antitumor immune responses. Several immune stimulatory genes display distinct expression patterns in the context of DDR deficiencies, suggesting their potential involvement in modulating immune surveillance. The observed positive correlation of *PVR* and *STAT1* expression with DDR mutations may reflect a compensatory upregulation of immune-activating pathways. In contrast, the negative correlation observed for other immune stimulatory genes may reflect the establishment of an immunosuppressive tumor microenvironment mediated by DDR alterations. Such immunological shifts may contribute to tumor immune escape and influence the efficacy of immune checkpoint blockade therapies. These results highlight the necessity for further mechanistic studies to elucidate how DDR gene mutations affect immune signaling networks and therapeutic responsiveness.

In the context of DNA Damage Repair (DDR) gene mutations, the immune inhibitor gene *IDO1* is positively correlated, showing increased expression when DDR genes are mutated. In contrast, several immune inhibitor genes, including *ADORA2A*, *CD160*, *CD244*, *HAVCR2*, *IL10RB*, *LGALS9*, *TGFB1*, and *TGFBR1*, were negatively correlated with DDR gene mutations, indicating a reduced expression under these conditions.

When DNA Damage Repair (DDR) genes are mutated, the MHC pathway-related gene *B2M* shows a negative correlation, indicating decreased expression. In contrast, *TAPBP* was also negatively correlated but with slightly less pronounced changes than *B2M*.

The changes in Z-scores between the non-mutated and mutated samples for each analysis are presented in Supplementary Table S17 of the supplementary data. Figure as Fig. 3 illustrates the impact of DNA damage repair gene mutations on the expression of immunostimulatory genes, including the following comparisons: *TP53* non-mutated versus mutated, *SMUG1* non-mutated versus mutated, *MGMT* non-mutated versus mutated, *MSH2* non-mutated versus mutated, *ERCC2* non-mutated versus mutated, *BRCA2* non-mutated versus mutated, *FANCA* non-mutated versus mutated, *XRCC6* non-mutated versus mutated, *NUDT1* non-mutated versus mutated, *POLQ* non-mutated versus mutated, *ENDOV* non-mutated versus mutated, *H2AX* non-mutated versus mutated, *SHPRH* non-mutated versus mutated, and *CDC25A* non-mutated versus mutated.

The mutational impact of DNA damage repair genes on the expression of MHC pathway-related genes is shown in Figure as Fig. 5. This included gene expression profiling of MHC pathway-related genes in non-mutated versus mutated samples from 14 DNA repair gene pathway categories, with one gene selected from each category. The categories for each gene are listed in Table 1. For example, *TP53* belongs to the “Other identified genes with known or suspected DNA repair function” category, *SMUG1* to the “Base excision repair (BER),” *MGMT* to the “Direct reversal of damage,” *MSH2* to the “Mismatch excision repair (MMR),” *ERCC2* to the “Nucleotide excision repair (NER),” *BRCA2* to the “Homologous recombination,” *FANCA* to the “Fanconi anemia—tolerance and repair of DNA crosslinks and other adducts,” *XRCC6* to the “Nonhomologous end-joining,” *NUDT1* to the “Modulation of nucleotide pools,” *POLQ* to the “DNA polymerases,” *ENDOV* to the “Editing and processing nuclease,” *H2AX* to the “Chromatin structure and modification,” *CDC25A* to the “Cell cycle checkpoints,” and *SHPRH* to the “Ubiquitination and modification”. The positive and negative correlation data for each analysis are listed in Supplementary Table S17.

## Discussion

Our clustering and gene annotation analyses provided key insights into the functional organization of DNA damage repair (DDR) pathways. By categorizing 264 DDR genes into 14 distinct functional clusters, we identified gene groups with shared biological roles, underscoring the coordinated and interconnected nature of DDR mechanisms. These results are depicted in Figure as Fig. 1c and detailed in Table 1. The validity of our clustering strategy was further supported through cross-referencing with previously published literature and the GeneCards database, which confirmed the biological relevance and accuracy of our classification. In addition, our analysis was extended to the classification of immune-related genes, where we employed the MCODE algorithm to cluster 66 genes into 10 categories based on functional similarities and dissimilarities. Clustering revealed that these immune-related genes were organized into distinct functional groups, with validation against published literature and databases supporting the accuracy of our classification. These findings emphasize the importance of identifying and understanding subnetworks within biological systems, as they can reveal critical proteins and complexes that may be pivotal for future biological and therapeutic research. By mapping these subnetworks, we can better understand key interactions that influence immune response, resistance mechanisms, and tumor progression, ultimately contributing to the development of more effective and personalized cancer treatment strategies. To obtain these insights, we analyzed the differential expression of immune-related genes associated with DDR gene mutations. A simple threshold approach was employed to determine the significance of changes in Z-scores between non-mutated and mutated samples, with a specific cutoff value (e.g., 0.5) used to flag significant changes. Several immune-related genes exhibited consistent differential expression across multiple DDR gene mutations. For instance, *PVR* was upregulated in response to mutations in 5 out of the 14 representative DDR genes. Similarly, *STAT1* expression was elevated in samples harboring mutations in several DDR genes. In contrast, *ENTPD1* was downregulated in 13 out of the 14 DDR gene mutation contexts, suggesting a broad suppression associated with DDR alterations. Additionally, *IL6R* and *STING1* were downregulated in response to mutations in 10 and 11 DDR genes, respectively. These findings highlight the potential immunomodulatory impact of DDR gene mutations. A comprehensive list of the mutated DDR genes and their corresponding immune-related gene expression profiles is written below and in Supplementary Table 17. Mutation profiling plays a crucial role in cancer biomarker discovery by identifying genetic alterations that drive tumorigenesis, influence treatment response, and serve as potential therapeutic targets. DDR genes, in particular, have been extensively studied for their role in genomic instability, a hallmark of cancer. Previous research, including pan-cancer genetic analyses of cuproptosis and copper metabolism-related gene sets (2022)^64^, cuproptosis gene sets (2022)^65^, disulfidptosis-related gene sets (2023)^66^, and mitotic intra-S DNA damage checkpoint genes (2024)^67^, has demonstrated how systematic mutation profiling can reveal key molecular signatures linked to cancer progression and therapeutic vulnerabilities. By integrating mutation profiling with immune regulatory gene expression analysis, this study aims to highlight the significance of DDR gene mutations as potential biomarkers for predicting immune response and guiding personalized cancer therapy.

These genes are affected by mutations in *TP53*,* SMUG1*,* MGMT*,* MSH2*,* ERCC2*,* BRCA2*,* FANCA*,* XRCC6*,* NUDT1*,* POLQ*,* ENDOV*,* H2AX*, and *SHPRH*. This recurrent pattern suggests that *IL6R* and *STING1* play central roles in the immune response associated with various types of DDR dysfunctions. *ENTPD1* (in the cell adhesion category) also shows differential expression across many DDR gene mutations, indicating its potential involvement in immune responses related to changes in DNA repair mechanisms. Liquid biopsy has emerged as a transformative tool in cancer diagnostics and monitoring, enabling non-invasive detection of tumor-specific genetic alterations. Recent advancements in circulating tumor DNA (ctDNA) detection have demonstrated its clinical utility in assessing treatment response, relapse, and recurrence, as highlighted in studies such as “Updates on liquid biopsies in neuroblastoma for treatment response, relapse, and recurrence assessment, 2024.”^68^ Furthermore, emerging sequencing technologies, including molecular barcode detection systems, have significantly improved the sensitivity and accuracy of DNA analysis, as demonstrated in “Development of a molecular barcode detection system for pancreaticobiliary malignancies and comparison with next-generation sequencing, 2024.”^69^ Methylation-based detection methods are also gaining prominence, with “Methylation signatures as biomarkers for non-invasive early detection of breast cancer: A systematic review of the literature, 2024”^70^ showcasing how DNA methylation profiling enhances early cancer detection. Given that DDR gene mutations contribute to genomic instability, their presence could potentially be detected through liquid biopsy techniques. By integrating ctDNA sequencing and methylation-based analysis, DDR-related alterations may serve as biomarkers for early cancer detection, prognosis, and therapeutic response monitoring. Advancements in these diagnostic approaches hold promise for refining precision oncology strategies and improving patient outcomes.

Although some genes are commonly affected by multiple DDR mutations, others exhibit unique differential expression patterns associated with specific DDR gene mutations. For example, *CD274* (immune inhibitory, co-inhibitor category) was uniquely differentially expressed only in the context of *SMUG1* mutations, suggesting a specific immunosuppressive response related to base excision repair pathway dysfunction. *CD40LG* and *CXCL12* (ligand category) were uniquely differentially expressed in *TP53* and *ENDOV* mutants, respectively, indicating that these ligands may be particularly sensitive to alterations in specific DDR pathways.

Furthermore, *TAPBP* (MHC pathway, antigen presentation category) was differentially expressed only in *POLQ* mutations, indicating the unique impact of *POLQ* mutations on antigen presentation. *HHLA2* (T-cell activation category) was observed only in *XRCC6* and *SHPRH* mutations, suggesting a specific role for these genes in modulating T-cell activation in response to DNA damage repair through nonhomologous end joining (NHEJ) and ubiquitination pathways. Finally, *TGFBR1* (immune inhibitory receptor) was differentially expressed in *MSH2* and *CDC25A* mutants, suggesting a specific link between these DDR genes and the modulation of immune inhibitory signals. To further validate these findings, future studies should explore patient-derived xenograft (PDX) models to assess the functional relevance of DDR gene mutations in regulating immune pathways. PDX models provide a robust platform for evaluating gene expression changes in a tumor microenvironment that closely mimics human cancer biology. Previous research, such as “Comparing volatile and intravenous anesthetics in a mouse model of breast cancer metastasis, 2018,”^71^ has demonstrated the utility of xenograft models in studying cancer progression and immune interactions. Investigating DDR-associated immune modulation in PDX models could help establish a mechanistic link between DDR mutations and immune regulatory gene expression, paving the way for potential therapeutic interventions targeting these pathways.

We observed a nuanced relationship between DNA Damage Repair (DDR) gene mutations and the regulation of immune-related genes. Specifically, *PVR* and *STAT1* demonstrated a positive correlation with increased expression in the presence of DDR gene mutations, suggesting that these genes may play an enhanced role in the immune response under mutational stress. Conversely, several immune stimulator genes, such as *CD40LG*, *CD48*, *CD86*,* CSF1R*,* CXCR4*,* ENTPD1*,* HHLA2*,* IL6R*,* KLRK1*,* PTEN*,* STING1*,* TMIGD2*,* TNFRSF14*,* TNFSF13*, and *TNFSF14*, were negatively correlated with DDR gene mutations, indicating the suppression of their expression. Similarly, among the immune inhibitors, *IDO1* showed a positive correlation with increased expression, whereas *ADORA2A*,* CD160*,* CD244*,* HAVCR2*,* IL10RB*,* LGALS9*,* TGFB1*, and *TGFBR1* were negatively correlated, reflecting a reduction in their expression. Furthermore, within the MHC pathway, *B2M* showed a pronounced negative correlation, suggesting decreased expression, whereas *TAPBP* exhibited a negative trend, albeit to a lesser extent. These findings underscore the complex interplay between DDR gene mutations and immune regulation, where certain genes are upregulated, whereas others are downregulated, potentially affecting the overall immune response and highlighting areas for further investigation.

These findings not only enhance our understanding of the complex interactions between DNA repair mechanisms and immune signaling pathways but also offer potential avenues for targeted therapeutic interventions in DDR-deficient tumors^26,14^. The consistency and specificity of immune gene responses to different DDR mutations underscores the potential for developing more precise and effective immunotherapies tailored to the genetic makeup of tumors.

It is important to acknowledge the potential biases associated with using TCGA datasets, which may influence the interpretation of our findings. TCGA, while a valuable resource, has inherent limitations due to technical and biological biases in bulk transcriptomic data, as discussed in “Genetic Expression in Cancer Research: Challenges and Complexity, 2024”^72^ and “Technical and Biological Biases in Bulk Transcriptomic Data Mining for Cancer Research, 2025^73^.” These biases may arise from sample heterogeneity, sequencing depth variations, and batch effects, potentially impacting gene expression correlations. To mitigate these limitations, future studies should consider integrating alternative datasets, such as single-cell RNA sequencing (scRNA-seq) or data from independent cohorts, to validate our findings and provide a more comprehensive understanding of the relationship between DDR mutations and immune gene regulation.

The Cancer Genome Atlas (TCGA) has been an invaluable resource in advancing cancer biomarker discovery, providing critical insights into the molecular underpinnings of various cancers. Our study builds upon the extensive body of research supported by TCGA, which has elucidated the role of specific biomarkers in cancer diagnosis and prognosis. Notable studies have demonstrated the potential of various biomarkers identified through TCGA analysis. For example, “Is the voltage-gated sodium channel β3 subunit *(SCN3B)* a biomarker for glioma? 2024” explores the role of *SCN3B* in glioma^74^, while “A Comprehensive Bioinformatic Analysis of Cyclin-dependent Kinase 2 *(CDK2)* in Glioma, 2022” highlights CDK2’s contribution to glioma progression^75^. Similarly, “Clinical powers of Aminoacyl tRNA Synthetase Complex Interacting Multifunctional Protein 1 *(AIMP1)* for head-neck squamous cell carcinoma, 2022”^76^ and “Potential roles of Cornichon Family AMPA Receptor Auxiliary Protein 4 *(CNIH4)* in head and neck squamous cell carcinoma, 2022” discuss the diagnostic and prognostic potential of *AIMP1* and *CNIH4* in head and neck squamous cell carcinoma^77^. Furthermore, "*RAD50* is a potential biomarker for breast cancer diagnosis and prognosis, 2024” demonstrates the relevance of *RAD50* in breast cancer^78^. These studies underscore the potential of TCGA data to identify biomarkers not only for specific cancer types but also for broader therapeutic applications.

By integrating DDR biomarker studies with findings from these key TCGA studies, we can enhance our understanding of the molecular alterations that drive tumorigenesis and immune regulation. This would not only improve the specificity of DDR biomarker identification but also inform personalized treatment strategies in cancer therapy.

## Conclusion

In this study, our comprehensive clustering and gene annotation analysis significantly advanced our understanding of DNA damage repair (DDR) pathways and their interactions with immune-related genes. By categorizing 264 genes into 14 distinct clusters and validating these groups against established databases and literature, we highlighted the intricate network of biological functions within the DDR pathways. Our approach not only confirmed the accuracy of the clustering but also revealed critical insights into how these pathways are interconnected.

The extension of our analysis to immune-related genes, in which we categorized the 66 genes into 10 functional clusters, further demonstrated the importance of subnetworks within biological systems. The consistent and distinct patterns of differential gene expression observed across various DDR gene mutations indicate that specific immunomodulatory genes, such as *IL6R* and *STING1*, may play pivotal roles in orchestrating the immune response to DDR dysfunction. These genes, along with others including *ENTPD1*, *CD274*, *TAPBP*, and *HHLA2*, represent promising candidates for further investigation as potential targets in immunotherapy and cancer treatment strategies.

These findings underscore the intricate effects of DDR gene mutations on immune regulation, suggesting both upregulation and downregulation of specific immune genes, which could have significant implications for understanding immune responses in the context of DNA damage and cancer. Further research is needed to explore these interactions and their potential impacts on therapeutic strategies.

Overall, our findings contribute to the growing body of knowledge on the relationship between DNA repair mechanisms and immune signaling. These findings provide a foundation for the development of more precise and effective immunotherapies tailored to the genetic profiles of DDR-deficient tumors. By identifying the key genes and pathways that respond to specific DDR gene mutations, this study opens up new avenues for targeted interventions in cancer treatment.

## Electronic supplementary material

Below is the link to the electronic supplementary material.

Supplementary Material 1

## Data Availability

The datasets analysed during the current study are available under the TCGA Pan-Cancer Atlas studies at https://www.cbioportal.org. Specific studies can be accessed by searching for “PanCancer Atlas” within cBioPortal. Additional gene-level information is available at https://www.genecards.org.
