# Supplementary material for: Pancancer analysis of DNA damage repair gene mutations and their impact on immune regulatory gene expression
Source: Sci Rep. 2025 May 5;15:15667. doi: 10.1038/s41598-025-99965-y (PMC12052996; doi:10.1038/s41598-025-99965-y)
Supplement: Supplementary file 1 — Supplementary Material 1 [file 41598_2025_99965_MOESM1_ESM.pdf]

Pancancer Analysis of DNA Damage Repair Gene Mutations and Their Impact on Immune Regulatory Gene Expression

Kanchana Yadav[1], Trishala Das[2], and Andrew M. Lynn[1]*

[1]School of Computational and Integrative Sciences, Jawaharlal Nehru University, New Delhi-110067, India

***Corresponding author:**

Prof. Andrew M. Lynn

School of Computational and Integrative Sciences,

Jawaharlal Nehru University,

New Delhi-110067, India

Email: andrew@jnu.ac.in

Supplementary Table S1. Shows the number of samples from each study

cancer sequence number	Cancer type by tissue	Cancer origin organ	Sample number in each cancer
1	Adrenocortical Carcinoma	Adrenal Gland	92 samples
2	Cholangiocarcinoma	Biliary Tract	36 samples
3	Bladder Urothelial Carcinoma	Bladder/Urinary Tract	411 samples
4	Colorectal Adenocarcinoma	Bowel	594 samples
5	Breast Invasive Carcinoma	Breast	1084 samples
6	Brain Lower Grade Glioma	CNS/Brain	514 samples
7	Glioblastoma Multiforme	CNS/Brain	592 samples

8	Cervical Squamous Cell Carcinoma	Cervix	297 samples
9	Esophageal Adenocarcinoma	Esophagus/Stomach	182 samples
10	Stomach Adenocarcinoma	Esophagus/Stomach	440 samples
11	Uveal Melanoma	Eye	80 samples
12	Head and Neck Squamous Cell Carcinoma	Head and Neck	523 samples
13	Kidney Renal Clear Cell Carcinoma	Kidney	512 samples
14	Kidney Chromophobe	Kidney	65 samples
15	Kidney Renal Papillary Cell Carcinoma	Kidney	283 samples
16	Liver Hepatocellular Carcinoma	Liver	372 samples
17	Lung Adenocarcinoma	Lung	566 samples
18	Lung Squamous Cell Carcinoma	Lung	487 samples
19	Diffuse Large B-Cell Lymphoma	Lymphoid	48 samples
20	Acute Myeloid Leukemia	Myeloid	200 samples
21	Ovarian Serous Cystadenocarcinoma	Ovary/Fallopian Tube	585 samples
22	Pancreatic Adenocarcinoma	Pancreas	184 samples
23	Mesothelioma	Pleura	87 samples
24	Prostate Adenocarcinoma	Prostate	494 samples
25	Skin Cutaneous Melanoma	Skin	448 samples

26	Pheochromocytoma and Paraganglioma	Soft Tissue	178 samples
27	Sarcoma	Soft Tissue	255 samples
28	Testicular Germ Cell Tumors	Testis	149 samples
29	Thymoma	Thymus	(123 samples
30	Thyroid Carcinoma	Thyroid	500 samples
31	Uterine Corpus Endometrial Carcinoma	Uterus	529 samples
32	Uterine Carcinosarcoma	Uterine Carcinosarcoma	57 samples

Supplementary Table 1 presents the 32 distinct human cancer types from The Cancer Genome Atlas (TCGA) PanCancer Atlas. The first column contains the serial numbers of these cancer types. The second column lists the cancer type based on the tissue of origin. The third column specifies the organ of origin for each cancer. The fourth column provides the total number of samples for each cancer type.

Supplementary Table S2. presents a list of genes involved in the base excision repair (BER) pathway.

**SN**	***Genes name***	**Gene ontology number**	**Repair Pathway**	**Evidence**	**PubMed IDs**
1	*UNG*	GO:0006284	base-excision repair	IEA	
2	*SMUG1*	GO:0006284	involved_in base-excision repair	IDA, IEA	
3	*TDG*	GO:0006284	involved_in base-excision repair	IDA, IEA	
4	*OGG1*	GO:0006284	involved_in base-excision repair	TAS, IEA	
5	*MUTYH*	GO:0006284	involved_in base-excision repair	IBA, IEA	

6	*NTHL1*	GO:0006284	base-excision repair	IEA	
7	*MPG*	GO:0006284	involved_in base-excision repair	IBA	
8	*NEIL1*	GO:0006284	involved_in base-excision repair	IDA, IBA, IEA	17611195
9	*NEIL2*	GO:0006284	involved_in base-excision repair	IBA	
10	*NEIL3*	GO:0006284	involved_in base-excision repair	IDA, ISS, IBA	
11	*APEX1*	GO:0006284	involved_in base-excision repair	IDA, IBA	8932386
12	*APEX2*	GO:0006284	involved_in base-excision repair	IBA	
13	*PNKP*	GO:0006287	involved_in base-excision repair, gap-filling	TAS	
14	*PARP2*	GO:0006284	involved_in base-excision repair	IEA	
15	*PARG*	GO:0006287	involved_in base-excision repair, gap-filling	TAS	
SN	***Genes name***		**Repair Pathway**		**confidence scores**
16	*MBD4*		Base-Excision Repair, AP Site Formation		0.89

The base excision repair genes were validated using Gene Ontology (GO) numbers, which are available on GeneCards (https://www.genecards.org/) and the published papers. The first column of the supplementary table contains the serial number of the genes. The second column lists the gene names. The third column provides the Gene Ontology (GO) number. The fourth column indicates the repair pathway in which the gene is involved. The fifth column presents the evidence supporting the association of these genes with the repair pathway. The sixth column includes the PubMed ID.

Supplementary Table S3. Show the genes involved in the direct reversal of damage

SN	***Genes name***	**Repair Pathway**	**confidence scores**
17	*MGMT*	MGMT-mediated DNA damage reversal	0.01
18	*ALKBH2*	ALKBH2 mediated reversal of alkylation damage	0.01
19	*ALKBH3*	ALKBH3 mediated reversal of alkylation damage	0.01
20	*ASCC3*	Reversal of alkylation damage by DNA dioxygenases	0.01

Supplementary Table 3 presents a list of genes involved in the direct reversal of damage. The repair pathways of these genes have been validated using the contained pathways available in GeneCards. The first column lists the serial numbers, the second column provides the gene names, the third column indicates the associated repair pathways, and the fourth column displays the confidence score.

Supplementary Table S4: Genes involved in mismatch excision repair (MMR)

**SN**	***Genes name***	**Gene ontology number**	**Repair Pathway**	**Evidence**	**PubMed IDs**
21	*MSH2*	GO:0006298	acts_upstream_of_or_within mismatch repair	IDA, IGI, IBA, IEA	23603115
22	*MSH3*	GO:0006298	involved_in mismatch repair	IDA, IMP, IBA, IEA	
23	*MSH6*	GO:0006298	involved_in mismatch repair	IDA, IMP, IGI, IBA, IEA	
24	*MLH1*	GO:0006298	acts_upstream_of_or_within mismatch repair	IGI, IBA, IEA	23603115

25	*PMS2*	GO:0006298	involved_in mismatch repair	IDA, IMP, IBA, TAS, IEA	
26	*PMS1*	GO:0006298	involved_in mismatch repair	IBA, IEA	
27	*MSH4*	GO:0006298	involved_in mismatch repair	IEA	
28	*MSH5*	GO:0006298	involved_in mismatch repair	IEA	
29	*MLH3*	GO:0006298	involved_in mismatch repair	IBA, NAS, IEA	12095912
30	*PMS2P3*	GO:0006298	involved_in mismatch repair	IEA	
31	*HMGB1*	GO:0032425	involved_in positive regulation of mismatch repair	IDA	15014079

Supplementary Table 4 presents the MMR genes validated using Gene Ontology (GO) terms, which are available on GeneCards (https://www.genecards.org/) and the published papers. The first column lists the serial numbers of the genes, the second column provides the gene names, the third column displays the Gene Ontology (GO) numbers, the fourth column indicates the repair pathway, the fifth column presents the evidence supporting the involvement of these genes in the mismatch repair pathway, and the sixth column contains the PubMed ID.

Supplementary Table S5. Presents the genes involved in the nucleotide excision repair (NER) pathway.

SN	***Genes name***	**Gene ontology number**	**Repair Pathway**	**Evidence**	**PubMed IDs**
32	*XPC*	GO:0006289	involved_in nucleotide-excision repair	IDA, ISS, IBA, TAS	33937266
33	*RAD23B*	GO:0006289	involved_in nucleotide-excision repair	IDA, TAS	8168482

34	*CETN2*	GO:0006289	involved_in nucleotide-excision repair	IDA, IBA	15964821
35	*RAD23A*	GO:0006289	involved_in nucleotide-excision repair	IDA, IEA	9372924
36	*XPA*	GO:0006289	involved_in nucleotide-excision repair	IDA, IEA	9372924
37	*DDB1*	GO:0006289	involved_in nucleotide-excision repair	TAS	
38	*DDB2*	GO:0006289	involved_in nucleotide-excision repair	IDA, ISS, TAS	33937266
39	*RPA1*	GO:0006289	involved_in nucleotide-excision repair	IMP, IBA, NAS	9430682
40	*RPA2*	GO:0006289	involved_in nucleotide-excision repair	IMP, IBA, NAS	9430682
41	*RPA3*	GO:0006289	involved_in nucleotide-excision repair	IMP, IBA, NAS	9430682
42	*ERCC2*	GO:0006283	involved_in transcription-coupled nucleotide-excision repair	IDA, TAS	
43	*GTF2H1*	GO:0006289	involved_in nucleotide-excision repair	IEA	
44	*GTF2H2*	GO:0006289	involved_in nucleotide-excision repair	IBA	
45	*GTF2H3*	GO:0006289	involved_in nucleotide-excision repair	IBA, IEA	
46	*GTF2H4*	GO:0006289	involved_in nucleotide-excision repair	IBA	
47	*GTF2H5*	GO:0006289	acts_upstream_of_or_within nucleotide-excision repair	IMP	23637614
48	*ERCC5*	GO:0006283	involved_in transcription-coupled nucleotide-excision repair	IMP, TAS	
49	*ERCC1*	GO:0000720	involved_in pyrimidine dimer repair by nucleotide-excision repair	IEA	

50	*ERCC4*	GO:0006289	involved_in nucleotide-excision repair	IDA, IMP	8797827
51	*ERCC8*	GO:0006283	acts_upstream_of_or_within transcription-coupled nucleotide-excision repair	IDA, IBA, IEA	12732143
52	*ERCC6*	GO:0006283	involved_in transcription-coupled nucleotide-excision repair	IMP, IBA, IEA	10564257
53	*UVSSA*	GO:0006283	involved_in transcription-coupled nucleotide-excision repair	IMP, IBA	22466610
54	*XAB2*	GO:0006283	involved_in transcription-coupled nucleotide-excision repair	IDA	10944529
55	*INO80*	GO:0010571	involved_in positive regulation of nuclear cell cycle DNA replication	IMP	20237820
56	*ERCC3*	GO:0000717	involved_in nucleotide-excision repair, DNA duplex unwinding	IMP	17466626
**SN**	***Genes name***		**Repair Pathway**		**confidence scores**
57	*TCEA1*		Nucleotide Excision Repair		0.77

The validation of this repair pathway was performed using GeneCards Gene Ontology annotations and the published papers. The corresponding Gene Ontology (GO) terms associated with NER genes are available on GeneCards (https://www.genecards.org/).

Supplementary Table S6. shows the genes involved in homologous recombination

SN	***Genes name***	**Gene ontology**	**Repair Pathway**	**Evidence**	**PubMed IDs**

		**number**			
58	*RAD51*	GO:0000724	involved_in double-strand break repair via homologous recombination	IDA, IMP, TAS	12427746
59	*RAD51B*	GO:0000724	involved_in double-strand break repair via homologous recombination	IMP, IBA, IEA	23149936
60	*RAD51D*	GO:0000724	involved_in double-strand break repair via homologous recombination	IMP, IBA	23149936
61	*SWI5*	GO:0000724	involved_in double-strand break repair via homologous recombination	IMP, IBA	21252223
62	*SPIDR*	GO:0000724	involved_in double-strand break repair via homologous recombination	IMP, IBA	23509288
63	*DMC1*	GO:0000724	double-strand break repair via homologous recombination	IEA	
64	*XRCC2*	GO:0000724	involved_in double-strand break repair via homologous recombination	IMP, IBA, IEA	23149936
65	*XRCC3*	GO:0000724	involved_in double-strand break repair via homologous recombination	IMP	23149936
66	*BRCA1*	GO:0000724	involved_in double-strand break repair via homologous recombination	IDA, IBA	17349954
67	*BARD1*	GO:0035825	involved_in homologous recombination	NAS	22369660
68	*SEM1*	GO:0000724	double-strand break repair via homologous	IEA	

			recombination		
69	*RAD50*	GO:0000724	involved_in double-strand break repair via homologous recombination	IDA	27889449
70	*NBN*	GO:0000724	involved_in double-strand break repair via homologous recombination	IDA, IBA	22464731
71	*RBBP8*	GO:0000724	involved_in double-strand break repair via homologous recombination	IDA, IMP	26721387
72	*SLX1A*	GO:0000724	involved_in double-strand break repair via homologous recombination	IMP, IBA	19595721
73	*SLX1B*	GO:0000724	involved_in double-strand break repair via homologous recombination	IMP, IBA	19595721
74	*GEN1*	GO:0000724	involved_in double-strand break repair via homologous recombination	IMP	23166748
75	*PPP4C*	GO:0000724	involved_in double-strand break repair via homologous recombination	IBA	
76	*PPP4R2*	GO:0010569	involved_in regulation of double-strand break repair via homologous recombination	IMP	20154705
77	*RTEL1*	GO:0010569	involved_in regulation of double-strand break repair via homologous recombination	IMP, IBA	18957201
78	*BLM*	GO:0000724	involved_in double-strand break repair via homologous recombination	IDA, IBA, NAS	11309417
79	*RMI1*	GO:0000724	involved_in double-strand break repair via	IDA, IBA	235437

			homologous recombination		48
80	*TOP3A*	GO:0000724	involved_in double-strand break repair via homologous recombination	IDA	23543748
81	*WRN*	GO:0000724	involved_in double-strand break repair via homologous recombination	IBA	
82	*RECQL4*	GO:0000724	involved_in double-strand break repair via homologous recombination	IBA	
83	*ATM*	GO:0000724	involved_in double-strand break repair via homologous recombination	IDA, IEA	10802669
84	*NABP2*	GO:0000724	involved_in double-strand break repair via homologous recombination	IDA, IMP, IBA	
85	*MORF4L1*	GO:0000724	involved_in double-strand break repair via homologous recombination	IDA	20332121
86	*RMI2*	GO:0000724	involved_in double-strand break repair via homologous recombination	IDA	23543748
87	*PARPBP*	GO:2000042	involved_in negative regulation of double-strand break repair via homologous recombination	IMP, IBA, IEA	22153967
88	*HELQ*	GO:0000724	involved_in double-strand break repair via homologous recombination	IMP	19995904
89	*SWSAP1*	GO:0000724	involved_in double-strand break repair via homologous recombination	IMP, IBA	21965664

90	*ZSWIM7*	GO:0000724	involved_in double-strand break repair via homologous recombination	IMP, IBA	21965664
91	*RAD52*	GO:0000724	involved_in double-strand break repair via homologous recombination	IBA	
92	*RAD54L*	GO:0000724	double-strand break repair via homologous recombination	IEA	
93	*RAD54B*	GO:0000724	acts_upstream_of_or_within double-strand break repair via homologous recombination	IDA, IBA	16428451
94	*SMC5*	GO:0000724	involved_in double-strand break repair via homologous recombination	IMP, IBA, NAS	16810316
95	*SMC6*	GO:0000724	involved_in double-strand break repair via homologous recombination	IBA, NAS	16810316
**SN**	***Genes name***		**Repair Pathway**		**confidence scores**
96	*BRCA2*		Defective HDR through Homologous Recombination Repair (HRR) due to PALB2 loss of BRCA1 binding function		0.93
97	*BRIP1*		Homologous DNA Pairing and Strand Exchange		0.93

98	*PALB2*		Defective HDR through Homologous Recombination Repair (HRR) due to PALB2 loss of BRCA1 binding function		0.93
99	*RAD51C*		Homologous DNA Pairing and Strand Exchange		0.93
100	*SLX4*		HDR through Homologous Recombination (HRR)		0.61
101	*MUS81*		HDR through Homologous Recombination (HRR)		0.61
102	*EME1*		HDR through Homologous Recombination (HRR)		0.61
103	*EME2*		HDR through Homologous Recombination (HRR)		0.61

Validation of the homologous recombination genes was performed using GeneCards and published papers. The GO term for the validation, PubMed ID, and pathway details are provided in the table above. Additional terms are available on GeneCards (https://www.genecards.org/).

Supplementary Table S7 shows the genes involved in Fanconi anemia, which play a role in the tolerance and repair of DNA crosslinks and other DNA adducts.

**SN**	***Genes name***	**Repair Pathway**	**confidence scores**
104	*FANCA*	Fanconi anemia pathway	0.41
105	*FANCB*	Fanconi anemia pathway	0.41
106	*FANCC*	Fanconi anemia pathway	0.41
107	*FANCD2*	Fanconi anemia pathway	0.41
108	*FANCE*	Fanconi anemia pathway	0.41
109	*FANCF*	Fanconi anemia pathway	0.41
110	*FANCG*	Fanconi anemia pathway	0.41
111	*FANCI*	Fanconi anemia pathway	0.41
112	*FANCL*	Fanconi anemia pathway	0.41
113	*FANCM*	Fanconi anemia pathway	0.41
114	*FAAP20*	Fanconi Anemia Pathway	0.34
115	*FAAP24*	Fanconi anemia pathway	0.41

116	*FAAP100*	Fanconi anemia pathway	0.41
117	*UBE2T*	Fanconi anemia pathway	0.41
118	*FAN1*	Fanconi anemia pathway	0.41

Validation of the Fanconi anemia pathway genes was performed using GeneCards and published papers. The GO term for the validation, PubMed ID, and pathway details are provided in the table above. Additional terms are available on GeneCards (https://www.genecards.org/).

Supplementary Table S8. Shows the genes involved in the nonhomologous end-joining pathway

**SN**	***Genes name***	**Gene ontology number**	**Repair Pathway**	**Evidence**	**PubMed IDs**
119	*XRCC6*	GO:0006303	involved_in double-strand break repair via nonhomologous end joining	IMP, IBA, TAS, NAS, IEA	15824061
120	*XRCC5*	GO:0006303	involved_in double-strand break repair via nonhomologous end joining	IDA, IMP, IBA, TAS, NAS	15824061
121	*PRKDC*	GO:0006303	involved_in double-strand break repair via nonhomologous end joining	TAS, NAS	
122	*LIG4*	GO:0006303	involved_in double-strand break repair via nonhomologous end joining	IDA, IMP, IGI, IBA, TAS	
123	*XRCC4*	GO:0006303	involved_in double-strand break repair via nonhomologous end joining	IDA, IMP, IBA, NAS	16439205

124	*DCLRE1C*	GO:0006303	involved_in double-strand break repair via nonhomologous end joining	IBA, TAS	
125	*NHEJ1*	GO:0006303	involved_in double-strand break repair via nonhomologous end joining	IDA, IMP, IBA, IEA	16439204
126	*POLM*	GO:0006303	involved_in double-strand break repair via nonhomologous end joining	IBA, TAS	
127	*DNTT*	GO:0006303	involved_in double-strand break repair via nonhomologous end joining	IBA	
128	*DCLRE1B*	GO:0006303	involved_in double-strand break repair via nonhomologous end joining	IBA	
129	*DCLRE1A*	GO:0006303	involved_in double-strand break repair via nonhomologous end joining	IBA	
130	*HMGB2*	GO:0006303	involved_in double-strand break repair via nonhomologous end joining	ISS	
131`	*APLF*	GO:0006303	involved_in double-strand break repair via nonhomologous end joining	IMP	23689425
132	*HMCES*	GO:0097681	involved_in double-strand break repair via alternative nonhomologous end joining	IEA	
133	*RNF168*	GO:0006303	involved_in double-strand break repair via nonhomologous end joining	IDA	27153538
134	*PARP3*	GO:2001034	involved_in positive regulation of double-strand break repair via	IDA	24598253

			nonhomologous end joining		
**SN**	***Genes name***		**Repair Pathway**		**confidence scores**
135	*TDP1*		Nonhomologous End-Joining (NHEJ)		0.61
136	*TDP2*		Nonhomologous End-Joining (NHEJ)		0.61

Validation of the Nonhomologous End-Joining (NHEJ) pathway genes was performed using GeneCards and published papers. The GO term for the validation, PubMed ID, and pathway details are provided in the table above. Additional terms are available on GeneCards (https://www.genecards.org/).

Supplementary Table S9. Shows the list of the genes involved in the modulation of nucleotide pools

**SN**	***Genes name***	**Gene ontology number**	**Repair Pathway**	**Evidence**	**PubMed IDs**
137	*NUDT1*	GO:0006152	involved_in purine nucleoside catabolic process	IDA	11139615
138	*DUT*	GO:0006139	involved_in nucleobase-containing compound metabolic process	TAS	1325640
139	*RRM2B*	GO:0055086	nucleobase-containing small molecule metabolic process	IEA	
140	*DNPH1*	GO:0006195	involved_in purine nucleotide catabolic process	TAS	
141	*NUDT15*	GO:0006195	involved_in purine nucleotide catabolic process	IMP	26878724

142	*NUDT18*	GO:0055086	involved_in nucleobase-containing small molecule metabolic process	TAS	

Validation of the genes involved in the modulation of nucleotide pools performed using GeneCards and published papers. The GO term for the validation, PubMed ID, and pathway details are provided in the table above. Additional terms are available on GeneCards (https://www.genecards.org/).

Supplementary Table S10. Shows the list of the genes involved in the DNA polymerases

SN	*Genes name*	Gene ontology number	Repair Pathway	Evidence	PubMed IDs
143	*POLA1*	GO:0006269	involved_in DNA replication, synthesis of primer	IDA	2175912
144	*POLB*	GO:0006261	involved_in DNA-templated DNA replication	TAS	8168825
145	*POLD1*	GO:0006261	involved_in DNA-templated DNA replication	IDA, IBA	20334433
146	*POLD2*	GO:0006271	involved_in DNA strand elongation involved in DNA replication	IBA	
147	*POLD3*	GO:0006271	involved_in DNA strand elongation involved in DNA replication	IBA	
148	*POLD4*	GO:0006261	involved_in DNA-templated DNA replication	IDA, IBA	20334433
149	*POLE*	GO:0006261	involved_in DNA-templated DNA replication	IDA	33051204
150	*POLE2*	GO:0042276	involved_in error-prone translesion synthesis	IBA	
151	*POLE3*	GO:0000122	involved_in negative regulation of transcription	IDA	18838386

			by RNA polymerase II		
152	*POLE4*	GO:0006261	involved_in DNA-templated DNA replication	IDA, IBA	33051204
153	*REV3L*	GO:0006261	involved_in DNA-templated DNA replication	IEA, TAS	
154	*MAD2L2*	GO:0000122	involved_in negative regulation of transcription by RNA polymerase II	IDA	19443654
155	*REV1*	GO:0042276	involved_in error-prone translesion synthesis	IBA, TAS	11485998
156	*POLG*	GO:0006261	involved_in DNA-templated DNA replication	IDA, TAS	10608893
157	*POLH*	GO:0006260	involved_in DNA replication	IEA	
158	*POLI*	GO:0000122	involved_in negative regulation of transcription by RNA polymerase II	IDA, TAS	
159	*POLQ*	GO:0006278	involved_in RNA-templated DNA biosynthetic process	IEA	
160	*POLK*	GO:0006260	involved_in DNA replication	IEA	
161	*POLL*	GO:0006260	involved_in DNA replication	NAS	10982892
162	*POLN*	GO:0006261	involved_in DNA-templated DNA replication	IEA	
163	*PRIMPOL*	GO:0006269	involved_in DNA replication, synthesis of primer	IEA	

Validation of the genes involved in the DNA polymerase activities performed using GeneCards and published papers. The GO term for the validation, PubMed ID, and pathway details are provided in the table above. Additional terms are available on GeneCards (https://www.genecards.org/).

Supplementary Table S11. Shows the list of the genes involved in the editing and processing nucleases

SN	*Genes name*	Gene ontology number	Repair Pathway	Evidence	PubMed IDs
164	*FEN1*	GO:0043137	involved_in DNA replication, removal of RNA primer	IDA	18995831
165	*TREX1*	GO:0006260	involved_in DNA replication	NAS, IEA	10391904
166	*TREX2*	GO:0006259	involved_in DNA metabolic process	IDA	11279105
167	*EXO1*	GO:0110025	involved_in DNA strand resection involved in replication fork processing	IMP	26240375
168	*APTX*	GO:0006139	nucleobase-containing compound metabolic process	IEA	
169	*ENDOV*	GO:0006281	involved_in DNA repair	ISS, IEA	
170	*DNA2*	GO:0000729	involved_in DNA double-strand break processing	IDA	21325134
171	*EXO5*	GO:0006259	DNA metabolic process	IEA	
172	*BCAS2*	GO:0000375	involved_in RNA splicing, via transesterification reactions	TAS	9731529
173	*PLRG1*	GO:0000398	involved_in mRNA splicing, via spliceosome	IDA, IBA,	23742842

				NAS, IC, IEA	
174	*TYMS*	GO:0006206	pyrimidine nucleobase metabolic process	IEA	
175	*NMNAT1*	GO:0009165	involved_in nucleotide biosynthetic process	IC	16118205
176	*SPO11*	GO:0000706	involved_in meiotic DNA double-strand break processing	IBA	

Validation of the genes involved in the editing and processing of the nucleases performed using GeneCards and published papers. The GO term for the validation, PubMed ID, and pathway details are provided in the table above. Additional terms are available on GeneCards (https://www.genecards.org/).

Supplementary Table S12. Shows the list of the genes involved in the chromatin structure and modification

SN	***Genes name***	**Gene ontology number**	**Repair Pathway**	**Evidence**	**PubMed IDs**
177	*H2AX*	GO:0000794	located_in condensed nuclear chromosome	IEA	
178	*CHAF1A*	GO:0006334	involved_in nucleosome assembly	IDA, IBA	14718166
179	*SETMAR*	GO:0000793	colocalizes_with condensed chromosome	IDA, IBA	18790802
180	*ATRX*	GO:0006306	obsolete DNA methylation	TAS	
181	*SMARCA4*	GO:0006337	involved_in nucleosome disassembly	IDA	8895581
182	*SMARCAD1*	GO:0006338	involved_in chromatin remodelling	IMP, NAS	2154930

					7
183	*SMARCC1*	GO:0006338	involved_in chromatin remodelling	IDA, NAS, HDA	1007820 7
184	*SOX4*	GO:0000785	part_of chromatin	ISA	
185	*PER1*	GO:0006338	involved_in chromatin remodelling	ISS	

Validation of the genes involved in the chromatin structure and modification performed using GeneCards and published papers. The GO term for the validation, PubMed ID, and pathway details are provided in the table above. Additional terms are available on GeneCards (https://www.genecards.org/).

Supplementary Table S13. Shows the genes involved in the ubiquitination and modification

**SN**	***Genes name***	**Gene ontology number**	**Repair Pathway**	**Evidence**	**PubMed IDs**
186	*RNF8*	GO:0006511	involved_in ubiquitin-dependent protein catabolic process	IDA, IBA	22266820
187	*RNF4*	GO:0043161	involved_in proteasome-mediated ubiquitin-dependent protein catabolic process	IMP	18408734
188	*UBE2V2*	GO:0000209	involved_in protein polyubiquitination	TAS	10089880
189	*UBE2N*	GO:0033182	obsolete regulation of histone ubiquitination	IMP	
190	*USP1*	GO:0035520	involved_in monoubiquitinated protein deubiquitination	IMP	20129063
191	*WDR48*	GO:1902525	involved_in regulation of protein	IEA	

			monoubiquitination		
192	*HERC2*	GO:0016567	involved_in protein ubiquitination	IDA, IBA, IEA	
193	*RBX1*	GO:0006511	involved_in ubiquitin-dependent protein catabolic process	IDA, IBA	17636018
194	*RAD18*	GO:0006513	involved_in protein monoubiquitination	IBA, IEA	
**SN**	***Genes name***		**Repair Pathway**		**confidence scores**
195	*UBE2A*		Antigen processing: Ubiquitination and Proteasome degradation		0.81
196	*UBE2B*		Antigen processing: Ubiquitination and Proteasome degradation		0.81
197	*SHPRH*		E3 ubiquitin ligases ubiquitinated target proteins		0.75
198	*HLTF*		E3 ubiquitin ligases ubiquitinated target proteins		0.75

Validation of the genes involved in the ubiquitination and modification performed using GeneCards and published papers. The GO term for the validation, PubMed ID, and pathway details are provided in the table above. Additional terms are available on GeneCards (https://www.genecards.org/).

Supplementary Table S14. Shows the other identified genes with known or suspected DNA repair functions

SN	*Genes name*	Gene ontology number	Repair Pathway	Evidence	PubMed IDs
199	*RECQL*	GO:0006268	involved_in DNA unwinding involved in DNA replication	IBA	
200	*RECQL5*	GO:0006268	involved_in DNA unwinding involved in DNA replication	IBA	
201	*PARP4*	GO:0006974	involved_in DNA damage response	NAS	10477748
202	*KAT5*	GO:0000724	involved_in double-strand break repair via homologous recombination	IDA, IBA, IEA	27153538
		GO:0006289	involved_in nucleotide-excision repair	IDA	32034146
203	*UIMC1*	GO:0006325	chromatin organization	IEA	
		GO:0007095	involved_in mitotic G2 DNA damage checkpoint signalling	IMP	17525340
		GO:0010212	involved_in response to ionizing radiation	IMP	17525340
204	*MMS19*	GO:0006281	involved_in DNA repair	IEA	
205	*HFM1*	GO:0032508	involved_in DNA duplex unwinding	IEA	
206	*PAXIP1*	GO:0006338	involved_in chromatin remodelling	IDA	17178841
207	*PDS5B*	GO:0006281 3	involved_in DNA repair	IBA	
208	*SHLD1*	GO:2000042	involved_in negative regulation of double-strand break repair via homologous recombination	IDA, IBA	29656893

		GO:2001034	involved_in positive regulation of double-strand break repair via nonhomologous end joining	IDA, IBA, NAS	29656893
209	*SHLD2*	GO:2000042	involved_in negative regulation of double-strand break repair via homologous recombination	IDA	29656893
		GO:2001034	involved_in positive regulation of double-strand break repair via nonhomologous end joining	IDA, NAS	29656893
210	*MRE11A*	GO:0000724	involved_in double-strand break repair via homologous recombination	IDA, IBA	15741314
		GO:0006303	involved_in double-strand break repair via nonhomologous end joining	IDA, IBA	9651580
211	*LIG3*	GO:0000724	involved_in double-strand break repair via homologous recombination	TAS	
		GO:0006287	involved_in base-excision repair, gap-filling	TAS	
212	*XRCC1*	GO:0006284	involved_in base-excision repair	IDA, IBA, IEA	34102106
		GO:0006303	involved_in double-strand break repair via nonhomologous end joining	IEA	
213	*PARP1*	GO:1905051	involved_in regulation of base-excision repair	IDA	34102106
		GO:1905168	involved_in positive regulation of double-strand break repair via homologous recombination	IDA	26344098

214	*PCNA*	GO:0006287	involved_in base-excision repair, gap-filling	IEA	
		GO:0006298	involved_in mismatch repair	IDA, IBA	11005803
215	*RAD9A*	GO:0000077	involved_in DNA damage checkpoint signalling	IMP, IEA	21659603
		GO:0000724	involved_in double-strand break repair via homologous recombination	IBA	
216	*TOPBP1*	GO:0000076	involved_in DNA replication checkpoint signalling	IDA	21482717
		GO:0000077	involved_in DNA damage checkpoint signalling	IDA	17575048
		GO:0000724	involved_in double-strand break repair via homologous recombination	IDA	26811421
217	*LIG1*	GO:0006284	involved_in base-excision repair	IDA	19589734
		GO:0006298	involved_in mismatch repair	TAS	
218	*PRPF19*	GO:0000077	involved_in DNA damage checkpoint signalling	IMP	24332808
		GO:0000209	acts_upstream_of_or_within protein polyubiquitination	IDA	11435423

		GO:0006303	involved_in double-strand break repair via nonhomologous end joining	IMP	18263876
219	*IDH1*	GO:0006979	involved_in response to oxidative stress	IEA	
220	*SPRTN*	GO:0006281	DNA repair	IEA	
221	*PARK7*	GO:0006281	involved_in DNA repair	IDA	28596309
222	*TP53*	GO:0006355	involved_in regulation of DNA-templated transcription	IDA, IMP, IEA	25417702
		GO:0006974	involved_in DNA damage response	IDA, IMP, IEA	24356969
		GO:0006977	involved_in DNA damage response, signal transduction by p53 class mediator resulting in cell cycle arrest	IMP	16213212
		GO:0006978	involved_in DNA damage response, signal transduction by p53 class mediator resulting in transcription of p21 class mediator	IDA, IMP	16479015
		GO:0008156	involved_in negative regulation of DNA replication	IEA	
		GO:0030330	involved_in DNA damage response, signal transduction by p53 class mediator	IDA, IMP	7958916
		GO:0031571	involved_in mitotic G1 DNA damage	IMP	7958916

			checkpoint signalling		
		GO:0042771	involved_in intrinsic apoptotic signalling pathway in response to DNA damage by p53 class mediator	IDA, IMP	16213212
		GO:0043504	involved_in mitochondrial DNA repair	IEA	
		GO:0043516	involved_in regulation of DNA damage response, signal transduction by p53 class mediator	IEA	
		GO:0045892	involved_in negative regulation of DNA-templated transcription	IDA, IMP, NAS	16492744
		GO:0045893	involved_in positive regulation of DNA-templated transcription	IDA, IMP, IEA	20096447
223	*TP53BP1*	GO:0000077	involved_in DNA damage checkpoint signalling	IBA	
		GO:0006303	involved_in double-strand break repair via nonhomologous end joining	IDA	23333306
		GO:0006325	involved_in chromatin organization	IEA	
		GO:2000042	involved_in negative regulation of double-strand break repair via homologous recombination	IDA	23333306

224	*RIF1*	GO:0031509	involved_in subtelomeric heterochromatin formation	ISS	
		GO:2000042	involved_in negative regulation of double-strand break repair via homologous recombination	IDA	23333306
		GO:2001034	involved_in positive regulation of double-strand break repair via nonhomologous end joining	IDA	28241136
225	*CLK2*	GO:0010212	involved_in response to ionizing radiation	IMP	20682768
226	*PTEN*	GO:0051091	involved_in positive regulation of DNA-binding transcription factor activity	IMP	20123964
227	*AEN*	GO:0010212	involved_in response to ionizing radiation	IDA	16171785
228	*ALKBH1*	GO:0035552	obsolete oxidative single-stranded DNA demethylation	IBA	
229	*GADD45A*	GO:0042770	involved_in signal transduction in response to DNA damage	IMP	20160708
230	*RFC1*		PCNA-Dependent Long Patch Base Excision Repair		0.81
			Nucleotide excision repair		0.76
231	*RFC2*		Homologous DNA Pairing and Strand Exchange		0.93

			PCNA-Dependent Long Patch Base Excision Repair		0.81
			Dual incision in TC-NER		0.90
232	*RFC3*		Homologous DNA Pairing and Strand Exchange		0.93
			PCNA-Dependent Long Patch Base Excision Repair		0.81
			Dual incision in TC-NER		0.90
233	*RFC4*		Homologous DNA Pairing and Strand Exchange		0.93
			PCNA-Dependent Long Patch Base Excision Repair		0.81
			Dual incision in TC-NER		0.90
234	*RFC5*		Homologous DNA Pairing and Strand Exchange		0.93
			PCNA-Dependent Long Patch Base Excision Repair		0.81
			Dual incision in TC-NER		0.90
235	*PPP4R1*		May play a role in regulation of cell division in		

			renal glomeruli.		

Validation of genes with known or suspected DNA repair functions performed using GeneCards and published papers. The GO term for the validation, PubMed ID, and pathway details are provided in the table above. Additional terms are available on GeneCards (https://www.genecards.org/).

Supplementary Table S15. List of the genes involved in the cell cycle checkpoints

SN	*Genes name*	Gene ontology number	Repair Pathway	Evidence	PubMed IDs
236	*CDC25A*	GO:0000082	involved_in G1/S transition of mitotic cell cycle	TAS	
237	*CDC25B*	GO:0000086	involved_in G2/M transition of mitotic cell cycle	IBA, TAS	12400006
238	*CDC25C*	GO:0000079	involved_in regulation of cyclin-dependent protein serine/threonine kinase activity	TAS	
239	*CDC5L*	GO:0000077	involved_in DNA damage checkpoint signalling	IMP	24332808
240	*CUL3*	GO:0000082	involved_in G1/S transition of mitotic cell cycle	TAS	8681378
241	*CUL5*	GO:0000082	involved_in G1/S transition of mitotic cell cycle	TAS	8681378
242	*H2AFX*	GO:0000077	involved_in DNA damage checkpoint signalling	IDA	17974976
243	*MNAT1*	GO:0000082	involved_in G1/S transition of mitotic cell	IEA	

			cycle		
244	*PLK3*	GO:0000082	involved_in G1/S transition of mitotic cell cycle	IMP	17264206
245	*RAD9B*	GO:0000076	involved_in DNA replication checkpoint signalling	IBA	
246	*WEE1*	GO:0000086	involved_in G2/M transition of mitotic cell cycle	TAS	
247	*MDC1*	GO:0000076	involved_in DNA replication checkpoint signalling	IDA	21482717
248	*CHEK2*	GO:0000077	involved_in DNA damage checkpoint signalling	TAS	9889122
249	*RPA4*	GO:0000077	involved_in DNA damage checkpoint signalling	IDA	19942684
250	*TELO2*	GO:2000003	involved_in positive regulation of DNA damage checkpoint	NAS	20810650
251	*TTK*	GO:0007094	involved_in mitotic spindle assembly checkpoint signalling	IBA, IEA	
252	*GADD45G*	GO:0051726	involved_in regulation of cell cycle	IBA, IEA	
253	*ABRAXAS1*	GO:0007095	involved_in mitotic G2 DNA damage checkpoint signalling	IMP	17525340
254	*BRCC3*	GO:0007095	involved_in mitotic G2 DNA damage checkpoint signalling	IMP, IEA	17525341

255	*CUL4A*	GO:0000082	involved_in G1/S transition of mitotic cell cycle	TAS	8681378
256	*ATR*	GO:0000077	involved_in DNA damage checkpoint signalling	IDA, IBA	14657349
257	*ATRIP*	GO:0000077	involved_in DNA damage checkpoint signalling	TAS, IEA	14657349
258	*RAD1*	GO:0000077	involved_in DNA damage checkpoint signalling	IMP, IBA, IEA	21659603
259	*RAD9A*	GO:0000076	involved_in DNA replication checkpoint signalling	IBA	
260	*RAD17*	GO:0000076	involved_in DNA replication checkpoint signalling	TAS	9660800
261	*CHEK1*	GO:0000077	involved_in DNA damage checkpoint signalling	IDA, IMP	19716789
262	*CCNH*	GO:2000045	involved_in regulation of G1/S transition of mitotic cell cycle	IDA	23622515
263	*CDK7*	GO:2000045	involved_in regulation of G1/S transition of mitotic cell cycle	IDA	23622515
264	*MPLKIP*		May play a role in maintenance of cell cycle integrity by regulating mitosis or cytokinesis. MPLKI_HUMAN,Q8TAP9		

Validation of genes involved in the cell cycle checkpoints performed using GeneCards and published papers. The GO term for the validation, PubMed ID, and pathway details are provided in the table above. Additional terms are available on GeneCards (https://www.genecards.org/).

The 67 immune stimulator-, inhibitor- and MHC pathway-related genes were classified into 10 categories on the basis of their specific functions. The function of each gene was subsequently validated via the use of the gene card database. Table 16. The validation table is the validation table of each immune-related gene.

Supplementary Table S16. Validation table of 67 immune-related genes

Category of genes based on functions	S.N	*Gene Names*	Genes Function
Receptor	1	*CD27*	Receptor specifically expressed at the surface of T cells which binds and is activated by its ligand *CD70/CD27L* expressed by B cells (PubMed:28011863).
	2	*CD40*	Receptor for *TNFSF5/CD40LG* (PubMed:31331973).
	3	*CTLA4*	Cytotoxic T-lymphocyte-associated granule serine protease 4,B7 costimulatory protein receptor for *CD80,CD86*,negative regulatory T-cell presenting costimulatory molecule, member of the immunoglobulin superfamily, with an association of the exon 3 microsatellite,106 bp allele with autoimmune disorders (conferring susceptibility to Graves disease and to thyroid associated orbitopathy but not to systemic lupus erythematosus), susceptibility gene for the celiac disease and for multiple sclerosis.
	4	*HAVCR2*	Receptor for phosphatidylserine (PtSer); PtSer-binding is calcium dependent.

	5	*ICOS*	Inducible T-cell Co-Stimulator *(ICOS)* is a receptor expressed on activated T cells. It binds to *ICOS* ligand (*ICOSL*) and plays a role in T-cell activation, differentiation, and the development of follicular helper T cells (Tfh).
	6	*IL2RA (CD122)*	Receptor for interleukin-2 and involved in the regulation of immune tolerance by controlling regulatory T cells (*TREGs*) activity.
	7	*PDCD1 (PD-1)*	Inhibitory receptor on antigen activated T cells that plays a critical role in induction and maintenance of immune tolerance to self (PubMed:21276005).
	8	*TIGIT*	T-cell Immunoreceptor with Ig and ITIM domains (*TIGIT*) is an inhibitory receptor expressed on T cells and NK cells. It binds to *CD155* and *CD112*, negatively regulating T-cell and NK cell activity.
	9	*TNFRSF14 (HVEM)*	Receptor for four distinct ligands: The TNF superfamily members *TNFSF14*/*LIGHT* and homotrimeric *LTA*/lymphotoxin-alpha and the immunoglobulin superfamily members *BTLA* and *CD160*, altogether defining a complex stimulatory and inhibitory signalling network (PubMed:10754304, 18193050, 23761635, 9462508).
	10	*TNFRSF18 (CD357, GITR)*	Tumor necrosis factor receptor superfamily, member 18,expressed in normal T lymphocytes from thymus, spleen, lymph nodes, involved in regulation of T-cell receptor-mediated cell death.
	11	*TNFRSF4*	Tumor necrosis factor receptor superfamily, member 4,tax-transcriptionally activated glycoprotein 1 receptor.
	12	*TNFRSF9 (CD137, 4-1BB)*	Tumor necrosis factor receptor superfamily, member 9,cell differentiation antigen *CD137* (4-1BB), expressed on B and T cells, inhibiting lymphocyte proliferation, activating apoptosis.

	13	*CD48*	Glycosylphosphatidylinositol (GPI)-anchored cell surface glycoprotein that interacts via its N-terminal immunoglobulin domain with cell surface receptors including 2B4/CD244 or CD2 to regulate immune cell function and activation (PubMed:12007789, 27249817).
	14	*KLRC1*	Natural killer cell lectin-like receptor subfamily C,member 1 with two alternatively spliced isoforms A (NKG2A),B (NKG2B) KLRC1
	15	*TGFBR1*	TGFBR1 is not a ligand but a receptor for TGFB1. It forms a receptor complex with TGFBR2 upon ligand binding, initiating intracellular signalling cascades that lead to the regulation of cell growth, differentiation, and immune responses.
	16	*CSF1R*	CSF1R is a receptor. It binds to ligands such as CSF1 (M-CSF) and IL-34, leading to the activation of signalling pathways that regulate the survival, proliferation, and differentiation of macrophages and other myeloid lineage cells.
	17	*CXCR4*	CXCR4 is a receptor. It binds to the chemokine ligand CXCL12 (SDF-1), which regulates the migration, homing, and retention of hematopoietic stem cells in the bone marrow. CXCR4 also plays a role in immune cell trafficking and cancer metastasis.
	18	*IL6R*	IL6R is a receptor that binds to the cytokine IL-6. Upon ligand binding, it associates with the gp130 coreceptor, triggering downstream signalling pathways involved in inflammation, immune response, and cell survival.
	19	*TNFRSF13 C (BAFF-R)*	BAFF-R is a receptor for BAFF (TNFSF13B) and plays a critical role in B-cell survival and maturation. This receptor is essential for the maintenance of the peripheral B-cell pool and the generation of long-lived plasma cells and memory B cells.
	20	*ADORA2A*	ADORA2A is a receptor for adenosine, which is produced from the breakdown of ATP by enzymes like CD39 and CD73.

	21	*TNFSF15*	Receptor for TNFRSF25 and TNFRSF6B.
	22	*CD244*	CD244 is an inhibitory receptor expressed on NK cells and some T cells. It interacts with CD48 on target cells and modulates the activity of NK cells, particularly in the context of recognizing cells with abnormal MHC class I expression. CD244's function is context dependent, as it can deliver both inhibitory and activating signals depending on the coreceptors involved.
Coinhibitor	23	*CD274 (PD-L1)*	The interaction with PDCD1/PD-1 inhibits cytotoxic T lymphocytes (CTLs) effector function (By similarity).
	24	*CD276 (B7-H3)*	May play a protective role in tumor cells by inhibiting natural-killer mediated cell lysis as well as a role of marker for detection of neuroblastoma cells.
	25	*PDCD1LG2*	Interaction with PDCD1 inhibits T-cell proliferation by blocking cell cycle progression and cytokine production (By similarity).
Ligands	26	*CD40LG*	Tumor necrosis factor (ligand) superfamily, member 5,CD40 ligand on B cell, expressed on vascular endothelial cell, smooth muscle cell,T cell, macrophage, involved in Ig class switching, potentially involved in pathogenic cytokine production in inflammatory bone disease.
	27	*CD70*	Expressed at the plasma membrane of B cells, it is the ligand of the CD27 receptor which is specifically expressed at the surface of T cells (PubMed:28011863, 28011864, 8387892).
	28	*TGFB1 (Transforming Growth Factor Beta 1)*	transforming growth factor, beta 1,152.6 kDa, homodimerization, expressed by fibroblast, platelet, monocyte, chondrocyte, osteoblasts, stimulating articular chondrocyte cell growth through MAPK3 activation, including the latency associated peptide (LAP), involved in the growth inhibition suppression by the complex Rb/Bog,

			may be implicated in susceptibility to atherosclerosis, bone diseases or various forms of cancer, regarding the presence of particular alleles at the locus.
	29	*LGALS9 (Galectin-9)*	Galectin-9 is a ligand for TIM-3 (HAVCR2) and plays a significant role in immune regulation. The interaction between Galectin-9 and TIM-3 induces T-cell apoptosis and exhaustion, contributing to the regulation of immune tolerance and chronic infection responses.
	30	*CXCL12*	While CXCL12 (also known as SDF-1) is primarily a chemokine rather than a receptor, it acts as a ligand for the CXCR4 and CXCR7 receptors. It is involved in the migration and homing of hematopoietic cells, as well as in cancer metastasis.
	31	*LTA (Lymphotoxin Alpha)*	LTA is a cytokine that binds to the TNFRSF1A and TNFRSF1B receptors. It plays a role in the regulation of immune responses, particularly in the development of lymphoid organs and the promotion of inflammation. LTA is involved in the cytotoxic activity of T cells and the formation of tertiary lymphoid structures in chronic inflammation.
	32	*TNFSF13 (APRIL)*	APRIL is a ligand that binds to receptors TACI (TNFRSF13B) and BCMA (TNFRSF17). It promotes B-cell survival, plasma cell maintenance, and immunoglobulin class switching. APRIL is involved in the regulation of B-cell homeostasis and antibody production.
	33	*STAT3*	STAT3 is actually a transcription factor rather than a ligand. It is activated by various cytokines, including IL-6, IL-10, and others. Once activated, STAT3 translocates to the nucleus, where it promotes the expression of genes involved in cell survival, proliferation, and immune tolerance.
Costimulators	34	*CD80*	Involved in the costimulatory signal essential for T-lymphocyte activation.

	35	*ICOSLG (B7-H2)*	Ligand for the T-cell-specific cell surface receptor ICOS. Acts as a costimulatory signal for T-cell proliferation and cytokine secretion; induces also B-cell proliferation and differentiation into plasma cells.
	36	*CD86*	Receptor involved in the costimulatory signal essential for T-lymphocyte proliferation and interleukin-2 production, by binding CD28 or CTLA-4.
	37	*TNFRSF13B (TACI)*	Receptor for TNFSF13/APRIL and TNFSF13B/TALL1/BAFF/BLYS that binds both ligands with similar high affinity. Mediates calcineurin-dependent activation of NF-AT, as well as activation of NF-kappa-B and AP-1.<br>Involved in the stimulation of B- and T-cell function and the regulation of humoral immunity. TR13B_HUMAN,O14836
	38	*TNFRSF17 (BCMA)*	Tumor necrosis factor receptor superfamily, member 17,B-cell maturation gene, expressed in terminal stages of B-cell differentiation, fused with IL2 in t(4;16)(q26;p13) translocation.
	39	*TNFRSF25 (DR3)*	TNFRSF25 is a receptor that binds to the ligand TL1A (TNFSF15). It plays a role in the costimulation of T cells, particularly in enhancing the proliferation and survival of effector and regulatory T cells.
	40	*TNFRSF8 (CD30)*	CD30 is a costimulatory receptor that binds to the CD30 ligand (CD30L/TNFSF8). It is involved in T-cell activation and plays a role in the regulation of immune responses, particularly in T-cell-mediated immunity.
	41	*TNFSF13B (BAFF)*	Cytokine that binds to TNFRSF13B/TACI and TNFRSF17/BCMA. TNFSF13/APRIL binds to the same 2 receptors. Together, they form a 2 ligands -2 receptors pathway involved in the stimulation of B- and T-cell function and the regulation of humoral immunity.

	42	*TNFSF14 (LIGHT)*	Cytokine that binds to TNFRSF3/LTBR. Binding to the decoy receptor TNFRSF6B modulates its effects. Acts as a ligand for TNFRSF14/HVEM (PubMed:10754304, 9462508).<br>Upon binding to TNFRSF14/HVEM, delivers costimulatory signals to T cells, leading to T-cell proliferation and IFNG production (PubMed:10754304).
	43	*TNFSF18 (GITRL)*	GITRL is the ligand for GITR (TNFRSF18) and plays a role in the costimulation of T cells.
	44	*PTEN*	phosphatase and tensin homologue, antagonizing signal transduction downstream of PI-3 kinase by dephosphorylating phosphatidylinositol-phosphate (PtdInsP), expressed in normal colon, tumor suppressor gene, modulating cell cycle progression and cell survival, negative regulator of cell interactions with the extracellular matrix, mutated in multiple advanced cancers (prostate and colorectal carcinoma, primary glioblastoma, renal cell carcinoma, breast and brain cancer, small cell lung cancer, squamous cell carcinoma of head and neck, sporadic follicular thyroid tumor,Cowden syndrome, melanoma and Bannayan Zonana syndrome, endometrial atypical hyperplasia, high grade astrocytoma, lymphoid neoplasms, laryngeal tumors), inversely correlated with AKF1 PTEN
	45	*STAT1*	signal transducer and activator of transcription 1,91 kDa, activated during monocyte to macrophage differentiation as an early transcription factor initially activated by adherence and then able to modulate the expression of functional genes such as ICAM1 and FCGR1 STAT1
	46	*STING1*	involved_in activation of innate immune response
	47	*CD273*	Involved in the costimulatory signal, essential for T-cell proliferation and IFNG production in a PDCD1-independent manner.

Antigen presentation	48	*LAG3*	May function as a ligand for MHC class II (MHC-II) on antigen-presenting cells (APC), promoting APC activation/maturation and driving Th1 immune response.
	49	*B2M*	Components of the class I major histocompatibility complex (MHC). Involved in the presentation of peptide antigens to the immune system.
	50	*TAP1*	ATP binding cassette superfamily, subfamily B (MDR/TAP), member 2,MHC-linked transporter associated with antigen processing 1,controlling MHC class I antigen presentation pathway, interacting with ABCB3 to form a functional peptide-transporting complex.
	51	*TAPBP (Tapasin)*	Involved in the association of MHC class I with transporter associated with antigen processing (TAP) and in the assembly of MHC class I with peptide (peptide loading).
	52	*RAET1E*	involved_in antigen processing and presentation of endogenous peptide antigen via MHC class I via ER pathway, TAP-independent
	53	*TAP2*	Similar to TAP1, TAP2 forms a heterodimer with TAP1, and together they transport peptides from the cytosol into the ER for loading onto MHC class I molecules. TAP2 is equally important in the antigen processing pathway.
	54	*MICB*	MICB is a stress-induced ligand that belongs to the MHC class I-like family and is recognized by the NKG2D receptor on natural killer (NK) cells and certain T cells. Although not directly involved in classical antigen presentation, MICB plays a role in immune surveillance by signalling to NK cells and cytotoxic T cells when cells are under stress, such as during infection or tumorigenesis. This can lead to the destruction of infected or transformed cells.
Cell adhesion	55	*CD96*	Promotes NK cell-target adhesion by interacting with PVR present on target cells.

	56	*NT5E*	involved_in leukocyte cell–cell adhesion
	57	*ENTPD1*	Involved in cell adhesion
		PVR	Mediates NK cell adhesion and triggers NK cell effector functions.
Immune suppression	58	*CD160*	CD160 is a glycoprotein that can transmit inhibitory signals to T cells and NK cells, reducing their cytotoxic activity and cytokine production.
	59	*IDO1*	Acts as a suppressor of antitumour immunity (PubMed:14502282, 23103127, 25157255, 25691885) and its limits the growth of intracellular pathogens by depriving tryptophan (PubMed:25691885).
	60	*IL10RB*	IL10RB is a component of the IL-10 receptor complex, necessary for IL-10 signalling. It pairs with IL10RA to form a receptor complex that transduces the anti-inflammatory signals of IL-10. Binding of IL-10 to its receptor initiates signalling pathways that suppress the expression of pro-inflammatory genes and promote immune tolerance. IL10RB is also a shared receptor subunit for other cytokines like IL-22, IL-26, and IL-28.
	61	*KDR*	KDR is a receptor for vascular endothelial growth factors (VEGFs) and plays a pivotal role in angiogenesis, the formation of new blood vessels. While its primary role is in the vascular system, KDR also contributes to immune suppression in the tumor microenvironment. VEGF signalling through KDR can inhibit the maturation of dendritic cells and promote the development of regulatory T cells (Tregs), thereby creating an immunosuppressive environment that allows tumors to evade immune detection.

T-cell activation	62	*HHLA2*	Primarily acts as a costimulatory molecule that enhances T-cell activation, proliferation, and cytokine production through its interaction with receptors like TMIGD2 on T cells. This interaction is essential for the full activation of T cells, particularly in the context of immune responses to pathogens and tumors.
	63	*KLRK1 (NKG2D)*	Functions as a critical receptor on CD8+ T cells and NK cells, enhancing their activation and cytotoxic function upon recognition of stress-induced ligands. This receptor plays a key role in immune surveillance and the elimination of infected or transformed cells.
Anti-inflammatory cytokines	64	*IL-10 (Interleukin-10)*	IL-10 is a key anti-inflammatory cytokine that is produced by various immune cells, including regulatory T cells (Tregs), macrophages, and dendritic cells. It plays a crucial role in controlling inflammation and promoting immune tolerance.
Differentiation of Th0 to Th2	65	*TMIGD2*	Plays a role in cell–cell interaction, cell migration, and angiogenesis. Through interaction with HHLA2, costimulates T cells in the context of TCR-mediated activation. Enhances T-cell proliferation and cytokine production via an AKT-dependent signalling cascade. TMIG2_HUMAN,Q96BF3
	66	*IL-6 (Interleukin-6)*	IL-6 is a multifunctional cytokine that can influence the differentiation of T cells, including Th2 cells.

Supplementary Table S17. Represents the changes in Z-scores between non-mutated and mutated samples for each analysis, along with the data on positive and negative correlations.

**Pathway of DDR genes**	**DDR genes**	**Immune stimula**	**Immune stimulator genes**	**Immune inhibitor genes**	**Immune inhibitor genes**	**MHC pathway related genes**	**MHC genes expression difference when**

		tor genes	expression difference when DDR gene mutated and non mutated (Z score)		expression difference when DDR gene mutated and non mutated (Z score)		DDR gene mutated and non mutated (Z score)
**Other identified genes with known or suspected DNA repair function**	TP53	CD40LG	-0.772946	ADORA2A	-0.873842	B2M	-0.824290
		CD48	-0.677637	CD160	-0.815179	TAPBP	-0.512452
		CD86	-0.619908	CD244	-0.726993		
		CSF1R	-0.918156	HAVCR2	-0.519143		
		CXCR4	-0.631514	IL10RB	-0.527816		
		ENTPD1	-1.198796	LGALS9	-0.957612		
		HHLA2	-0.591752	TGFB1	-0.557681		
		IL6R	-0.945154				
		KLRK1	-0.827807				
		PVR	0.612378				
		PTEN	-0.872895				

		STING1	-1.494345			
		TMIGD2	-0.534667			
		TNFRSF14	-1.403395			
		TNFSF13	-1.738477			
		TNFSF14	-0.759198			
**Base excision repair (BER)**	SMUG1	ENTPD1	-0.705048	ADORA2A	-0.513480	
		IL6R	-0.901962	CD274	-0.529855	
		NT5E	-0.939494	TGFB1	-0.843212	
		STING1	-0.873289			
**Direct reversal of damage**	MGMT	ENTPD1	-0.890609	TGFB1	-0.521122	
		IL6R	-0.513871			
		STING1	-0.932606			
		TNFRSF14	-0.718613			

**Mismatch excision repair (MMR)**	MSH2	CSF1R	-0.508089	ADORA2A	-0.651162	
		ENTPD1	-1.051551	KDR	-0.611002	
		IL6R	-1.039827	TGFB1	-0.885390	
		NT5E	-0.745683	TGFBR1	-0.600722	
		PVR	0.756492			
		PTEN	-0.698969			
		STAT1	0.508839			
		STING1	-1.259975			
		TNFRSF14	-0.661890			
**Nucleotide excision repair (NER)**	ERCC2	ENTPD1	-0.952137			
		IL6R	-0.679924			
		NT5E	-0.644332			
		PVR	1.210907			
		STING1	-1.025833			
		TNFRSF14	-0.856266			

**Homologous recombination**	BRCA2	CD40	-0.554083	TGFB1	-0.616035		
		ENTPD1	-0.761711				
		IL6R	-0.723116				
		STING1	-0.567628				
		TNFRSF14	-0.634167				
**Fanconi anaemia-Tolerance and repair of DNA crosslinks and other adducts in DNA**	FANCA	CD40	-0.594095				
		ENTPD1	-0.740288				
		NT5E	-0.535492				
**Nonhomologous end-joining**	XRCC6	CD40	-0.587836	ADORA2A	-0.509352	B2M	-0.552105
		CSF1R	-0.514906	CD160	-0.533095		
		ENTPD1	-1.022129	TGFB1	-0.793946		
		HHLA2	-0.664743				

		IL6R	-0.802397				
		PVR	0.682354				
		PTEN	-0.577640				
		STAT1	0.966706				
		STING1	-0.999495				
		TNFRSF14	-0.756688				
**Modulation of nucleotide pools**	NUDT1	CD40	-0.553123	ADORA2A	-0.54073		
		ENTPD1	-0.802751	LGALS9	-0.52867		
		STAT1	0.607553	TGFB1	-0.56842		
		STING1	-0.820622				
		TNFRSF14	-0.715577				
		TNFSF13	-0.711261				
**DNA polymerases**	POLQ	CD40	-0.588010	ADORA2A	-0.843280	B2M	-0.716369
		CSF1R	-0.606046	IL10RB	-0.524176	TAPBP	-0.570899

		ENTPD1	-0.916674	KDR	-0.553201	
		IL6R	-0.611688	LGALS9	-0.515571	
		STING1	-1.369789	TGFB1	-0.708858	
		TNFRSF14	-0.966246			
		TNFSF13	-0.884272			
**Editing and processing nucleases**	ENDOV	CXCL12	-0.764122			
		ENTPD1	-0.548890			
		IL6R	-0.629474			
		PTEN	-0.549132			
		STING1	-0.545475			
		TNFRSF14	-0.857686			
		TNFRSF8	-0.579900			
		TNFSF13	-0.666453			

		TNFSF14	-0.511008				
**Chromatin Structure and Modification**	H2AX	CD40	-0.787652			B2M	-0.502231
		IL6R	-0.535386				
		STING1	-0.536029				
		TNFRSF14	-0.845959				
**Cell cycle Checkpoints**	CDC25A	ENTPD1	-0.559956	IDO1	0.568734		
		PVR	0.602594	PDCD1	0.533344		
				TGFBR1	-0.642428		
				ADORA2A	-0.648246		
				TGFB1	-0.575678		
**Ubiquitination and modification**	SHPRH	ENTPD1	-0.840107				

Immune stimulator, inhibitor, and MHC pathway-related genes that show a positive upregulation and downregulation. The positive value shows the upregulation and the negative value shows the downregulation.
